# Clinical magnetocardiography: the unshielded bet—past, present, and future

**DOI:** 10.3389/fcvm.2023.1232882

**Published:** 2023-08-10

**Authors:** D. Brisinda, P. Fenici, R. Fenici

**Affiliations:** ^1^ Dipartimento Scienze dell'invecchiamento, ortopediche e reumatologiche, Fondazione Policlinico Universitario Agostino Gemelli, IRCCS, Rome, Italy; ^2^ School of Medicine and Surgery, Catholic University of the Sacred Heart, Rome, Italy; ^3^ Biomagnetism and Clinical Physiology International Center (BACPIC), Rome, Italy

**Keywords:** magnetocardiography (MCG), electrophysiology arrhythmias mapping and ablation, source localization accuracy, inverse problem, electroanatomical imaging, magnetic sensors, gradiometer array, magnetically shielded room (MSR)

## Abstract

Magnetocardiography (MCG), which is nowadays 60 years old, has not yet been fully accepted as a clinical tool. Nevertheless, a large body of research and several clinical trials have demonstrated its reliability in providing additional diagnostic electrophysiological information if compared with conventional non-invasive electrocardiographic methods. Since the beginning, one major objective difficulty has been the need to clean the weak cardiac magnetic signals from the much higher environmental noise, especially that of urban and hospital environments. The obvious solution to record the magnetocardiogram in highly performant magnetically shielded rooms has provided the ideal setup for decades of research demonstrating the diagnostic potential of this technology. However, only a few clinical institutions have had the resources to install and run routinely such highly expensive and technically demanding systems. Therefore, increasing attempts have been made to develop cheaper alternatives to improve the magnetic signal-to-noise ratio allowing MCG in unshielded hospital environments. In this article, the most relevant milestones in the MCG's journey are reviewed, addressing the possible reasons beyond the currently long-lasting difficulty to reach a clinical breakthrough and leveraging the authors’ personal experience since the early 1980s attempting to finally bring MCG to the patient's bedside for many years thus far. Their nearly four decades of foundational experimental and clinical research between shielded and unshielded solutions are summarized and referenced, following the original vision that MCG had to be intended as an unrivaled method for contactless assessment of the cardiac electrophysiology and as an advanced method for non-invasive electroanatomical imaging, through multimodal integration with other non-fluoroscopic imaging techniques. Whereas all the above accounts for the past, with the available innovative sensors and more affordable active shielding technologies, the present demonstrates that several novel systems have been developed and tested in multicenter clinical trials adopting both shielded and unshielded MCG built-in hospital environments. The future of MCG will mostly be dependent on the results from the ongoing progress in novel sensor technology, which is relatively soon foreseen to provide multiple alternatives for the construction of more compact, affordable, portable, and even wearable devices for unshielded MCG inside hospital environments and perhaps also for ambulatory patients.

## 1. Introduction

Magnetocardiography (MCG) is a technique used for measuring the magnetic field (MF) produced by the electrical activity of the heart. Unlike electrocardiography (ECG), which measures the electrical activity of the heart indirectly using electrodes placed on the skin, MCG is a contactless recording of the magnetic fields produced by the electrophysiological activity inside the heart using highly sensitive magnetic sensors placed outside the body. Another advantage of MCG is that the recording of cardiac MF is not significantly affected by the different conductivity and electrical resistance of the various tissues interposed between the cardiac source and the surface sensors.

As compared with ECG, a foundational research from the general theory of bioelectromagnetism describes the amount of potential additional information provided by MCG (1–7), stimulating bio-physicists to find ways to effectively measure cardiac MF. These attempts were finally achieved in the early 1960s, experimentally by recording the cardiac MF from an isolated rabbit heart preparation with a toroidal solenoid (8) and in humans by Baule and McFee (9), using two large magnetic sensor coils, although at the very low spatial resolution, which obviously made it still inadequate for any practical use, especially looking for MCG as a potential diagnostic tool in the clinical setting. Moreover, a major limiting factor was that the cardiac magnetic field's strength is very weak (in the range of 10^−12^ to 10^−15^ T) compared with the Earth's MF (in the order of magnitude of 10^−6^ T); thus, MCG signals were strongly affected by environmental noise and practically useless compared with much easier recordable ECG. Baule and McFee tried to address this problem, using the sensor coil prototype arranged in a sort of “gradiometer” configuration, needing anyway the recording to happen in a “quiet” ideal location far from any potential magnetic interference.

Providing a significant improvement in sensitivity and spatial resolution of MF measurements, a major milestone in MCG was the invention of the first superconducting quantum interference device (SQUID) (10) and the installation of SQUID within the magnetically shielded room (MSR) of the Massachusetts Institute of Technology (MIT) that radically reduce external electromagnetic interference by at least a factor of about 1,000 (11, 12), finally providing an experimental setup and a magnetic signal-to-noise ratio (SNR) suitable for recording cardiac and even brain biomagnetic signals (13, 14). Since then, the research work of a few pioneers set the theoretical basis for cardiac MF interpretation and explored several potential applications of MCG as a reliable method to improve non-invasive diagnosis of cardiac abnormalities (1, 3, 15–21).

Although electromagnetic shielding (EMS) provided the best SNR, increasing the reliability and accuracy of MCG (22), highly efficient MSRs were very expensive, not easy to install everywhere, and especially not suitable in the clinical environment. Thus, aiming for clinical applications of magnetocardiography at scale, it became evident that cheaper and simpler alternatives were needed to dampen electromagnetic noise in clinical settings, which are well-known magnetically noisy environments.

To avoid the need for MSRs, the first technological alternative was the invention of superconducting pick-up coils designed as second- or higher-order gradiometers that would primarily measure the MF gradient closest to the source ignoring magnetic fields from further away (e.g., the environmental noise) (23). The efficacy of such a less expensive unshielded approach enhanced the opportunity to get more scientists involved in the field of biomagnetism research (24–32) and to attempt the first installation of a single-channel MCG system in a standard hospital unshielded room, testing and validating the potential use of MCG as a diagnostic tool “to the patient's bedside,” in a minimally adapted unshielded cardiology lab designed for simultaneous MCG and clinical interventional electrophysiological study (27, 33–35).

At that time, only single-channel devices were available, and the normal component of cardiac MF had to be sequentially measured at different locations in front of the chest, typically in a rectangular normalized (e.g., the “Finnish”) grid (24, 25). An alternative approach to single-channel mapping came from Stanford, where the three orthogonal components of cardiac MF (so-called vector magnetocardiography) were measured at a single position over the heart, based on the theoretical assumption that like in vector electrocardiography, the three-dimensional (3D) motion of the magnetic heart vector represents the overall activity of the heart (2, 36–38).

Another crucial step forward was the development of multichannel SQUID devices, allowing simultaneous multipoint mapping of the cardiac MF, which was absolutely needed for more reliable and precise real-time detection of its dynamic variation due to transient normal and/or abnormal electrophysiological events, such as acute myocardial ischemia or arrhythmias.

Since then, the history of magnetocardiography has become somehow complex and slowed down, with a progressive reduction of MCG scientific production presented at the biannual biomagnetism conferences, as low as less than 10 abstracts at the Biomag 2022 Conference in Birmingham (39). The alternating phases of clinicians’ skepticism and renewed enthusiasm (40) were often driven by the different perspectives among basic scientists, mostly favoring the development of huge and much more expensive installations in MSRs to guarantee optimal sensitivity and 24/7 reliability of MCG measurements but “de facto” far from clinicians’ needs seeking for a new diagnostic tool to the patient's bedside instead. Indeed, since the 1980s, multichannel installation in highly performant MSRs was the most preferred choice also for MCG, under the influence of faster-growing research and development for clinical applications of magnetoencephalography (MEG) (26, 41), which diverged major investments in that direction. In fact, although MEG feasibility required heavy and expensive EMS, its development was favored by the tremendous impact that neuromagnetism provided for the non-invasive functional imaging of brain electrophysiology compared with the huge limitation of the electric counterpart available at that time (42–46). Consequently, only a few centers of excellence working with innovative shielded multi-SQUID systems, mainly driven by bio-physicists and somewhere in time-sharing with neurology, continued basic MCG research (44–47) and several studies of clinical interest in collaboration with cardiology departments (48–65).

On the other hand, most clinicians were skeptical and considered unnecessary the sophisticated and expensive MCG technology, given the ready availability of more affordable, and overall well-established diagnostic tools (although mostly invasive) for cardiac clinical electrophysiology. Only a small group of “MCG believers” envisioning the yet unleashed innovative diagnostic power of this technology (if made available to the patient's bedside) devoted their main research focus to the development and validation of less expensive and more scalable multichannel MCG mapping systems, reliably operated in unshielded hospital environments (66–78). This pioneering vision of MCG mapping as a unique novel method for non-invasive 3D electroanatomical imaging (EAI) and localization of cardiac electrophysiological mechanisms with its potential to guide “aimed” myocardial biopsy and interventional transcatheter treatment of arrhythmogenic substrates prompted the development of a novel and easy-to-use unshielded MCG multichannel prototype reliable even in catheterization laboratories where noisy radiological and interventional equipment were necessary (79).

The parallel research efforts carried out with shielded and unshielded MCG over the last three decades have enlarged the knowledge about the pros and cons of these two approaches and provided evidence of well-defined fields of clinical application, such as the emergency triage of patients with chest pain, the diagnosis of different kinds of ischemic and non-ischemic cardiomyopathies, the heart transplant rejection (80, 81), the non-invasive 3D EAI of arrhythmogenic substrates and mechanisms (82), fetal MCG, and in particular the prenatal diagnosis of arrhythmogenic risk and cardiomyopathies (54, 83). Moreover, MCG has proven useful for the non-invasive study of experimental intact animals (84) and contactless high-resolution investigation of experimental electrophysiology of isolated heart (85) and cardiac tissue models (86).

Aside from a large body of relevant research and meta-analysis papers, there are also several comprehensive reviews, Biomag Conference Proceedings, and book chapters summarizing the history and the results of decades of experimental and clinical MCG research, as well as the major technological advancements obtained with shielded and unshielded settings (44–46, 82, 87–105).

Since the late 1970s, our group worked to bring MCG to the patient's bedside and to use it as a diagnostic tool in unshielded hospital environments (27, 33). Thus, we will focus on providing a review of the most relevant steps of MCG starting from our experience in developing devices and protocols for experimental and clinical validation of unshielded MCG as an unrivaled method for contactless non-invasive cardiac functional electrophysiological imaging, discussing such achievements in the light of gold standard MCG measurements carried out in MSRs in collaboration with other biomagnetism centers of excellence. Finally, we will also provide our personal vision of the future of MCG in light of present and foreseen improvements in magnetic sensor technology, innovative methods for signal processing, and less expensive active shielding approaches.

## 2. Unshielded MCG: the past

**Figure 1:**
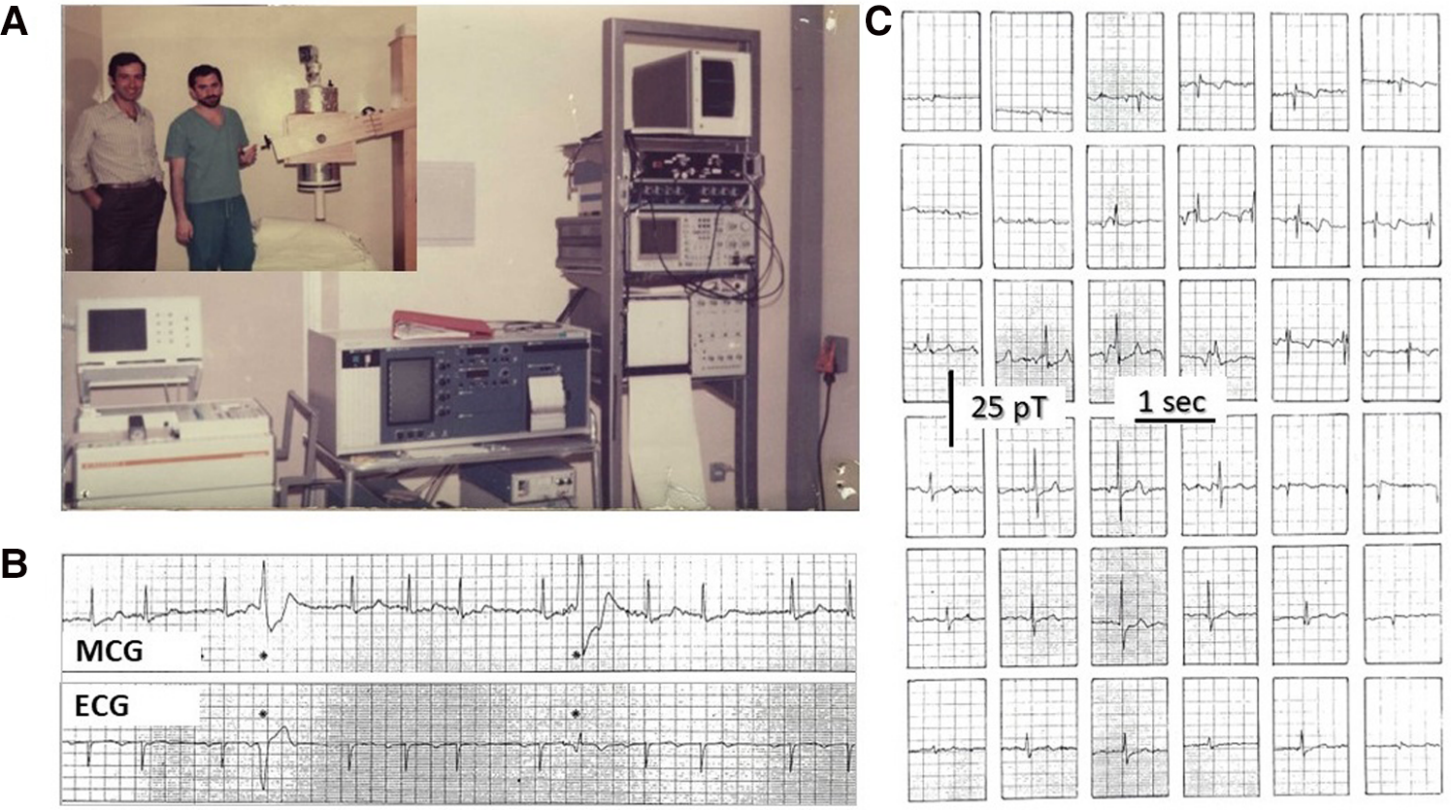
(**A**) CNR's physicist Gian Luca Romani (left) and Riccardo Fenici (right) testing the prototype of the CNR unshielded MCG SQUID gradiometer at the Catholic University's Gemelli Hospital in Rome. (**B**) Example of real-time simultaneous MCG and ECG recordings and (**C**) example of 36-position real-time single-beat MCG recordings (Finnish grid) [modified from (98)].

Indeed, MCG was born unshielded (8, 9, 15, 21). With the exception of the MIT (106–108), most MCG research in the early 1970s was attempted in very low-noise rural laboratories, wooden cottages, or underground locations, using first-, second- or higher-order gradiometers as pick-up coils, investigating healthy subjects and pregnancy as well as patients with cardiac disorders (6, 24, 25, 27, 29, 109–114). Although more susceptible to external electromagnetic interference and somehow less sensitive, unshielded MCG devices had the advantage of being less expensive and theoretically portable to the patient's bedside. Such potential was originally tested at the Catholic University Hospital of Rome in January 1980 by bringing the single-channel MCG prototype, designed and built by the researchers of the Italian National Research Council's Institute for Solid State Electronics (CNR—Consiglio Nazionale delle Ricerche—Istituto di Elettronica dello Stato Solido), in a standard hospital room of the Policlinico Gemelli and providing the first demonstration that its sensitivity was good enough to record beat-to-beat MCG in an unshielded clinical environment (Figure 1).

**Figure 2:**
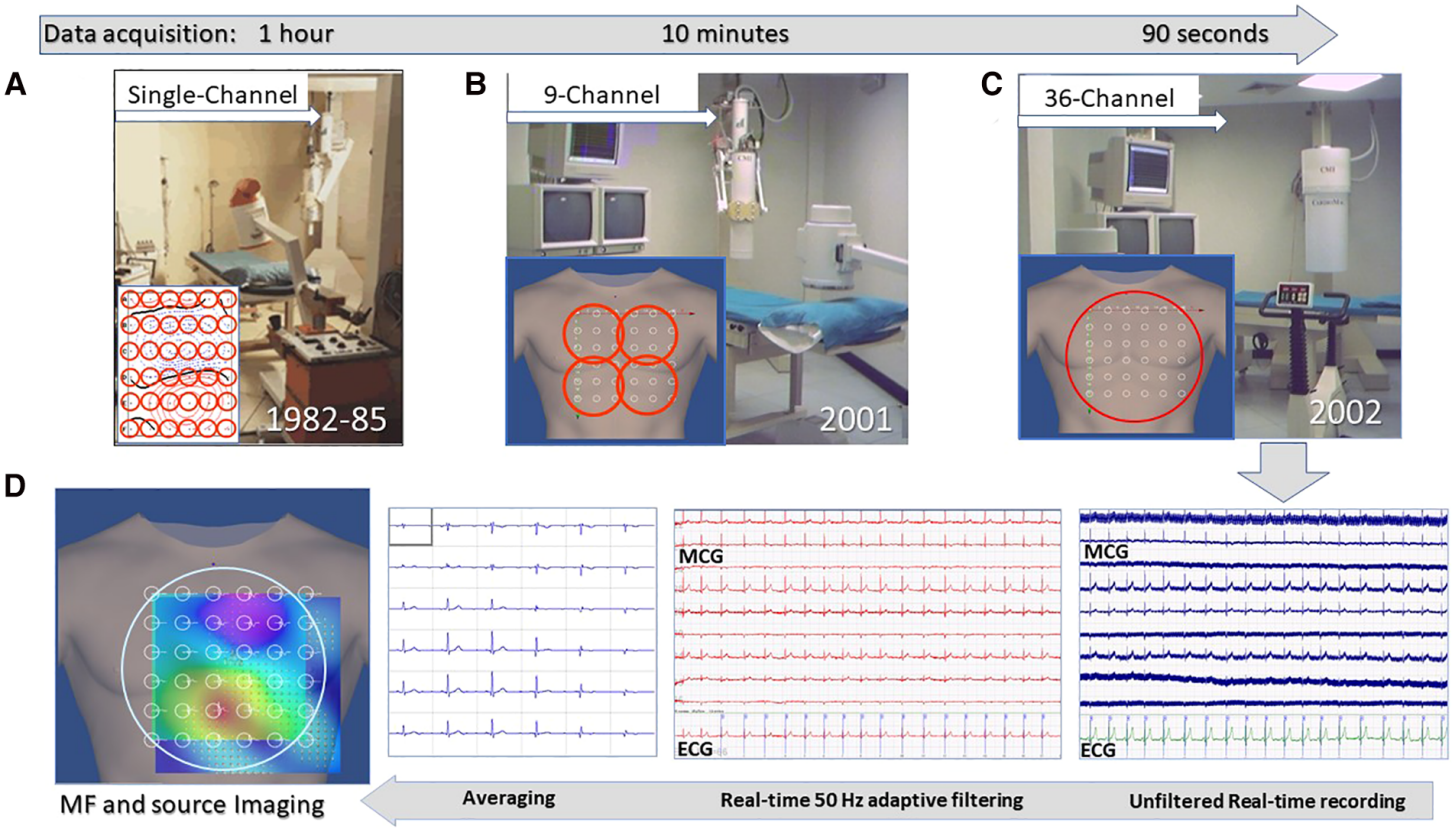
Unshielded magnetocardiography at the Catholic University Hospital's BACPIC: (**A**) Single-channel system; (**B**) Nine-channel CMI prototype; (**C**) CMI 3619a 36-channel; and (**D**) examples of real-time MCG recordings and of the signal processing flow chart for MF imaging and 3D source localization.

**Figure 3:**
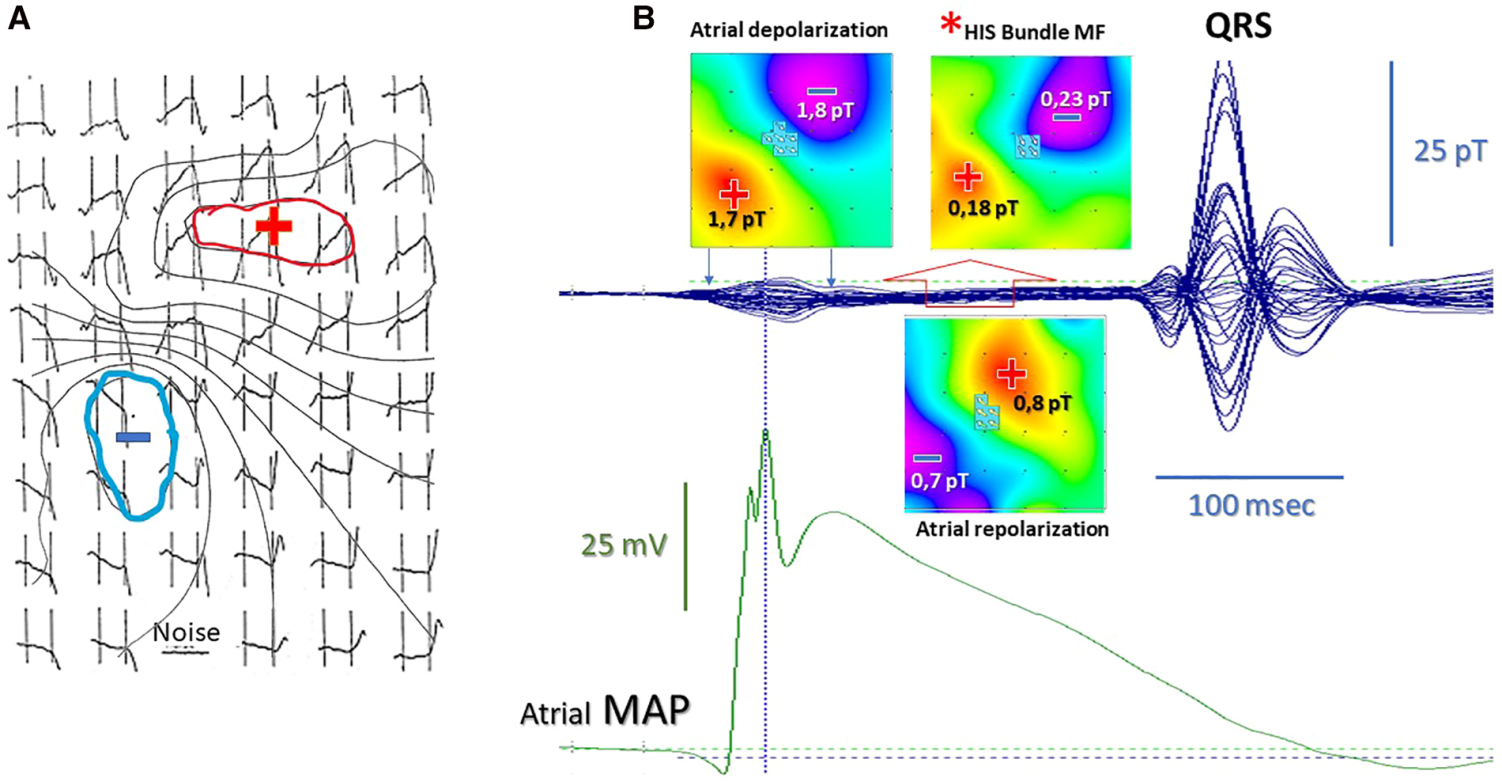
(**A**) High-resolution MCG map of the “ramp-like” pattern during the PR interval with superimposed isomagnetic lines [modified from (120) data]. (**B**) Validation of the atrial repolarization nature of the PR “ramp-like” MCG pattern with simultaneous MCG and atrial monophasic action potential recording. The red asterisk indicates the His bundle MF (±0.2pT) disclosed during the last half of the PR interval after subtraction of the stronger (±1.8 pT) atrial repolarization MF (open red arrow). The pseudo-current reconstruction (multiple white arrows on the MF maps) indicates the opposite direction of atrial and His bundle depolarization current and of atrial repolarization current [standardized MF color code: in blue (−), MF outgoing from the chest; in red (+), MF entering in the chest].

The CNR's prototype was later replaced by an industrialized single-channel system in 1982 (Elettronica SpA, Rome) (Figure 2A). With that system and new software tools for digital data acquisition and signal averaging, it was possible to detect even the ultra-weak magnetic fields generated during the ECG PR interval to attempt non-invasive MCG detection of the His bundle signal (115). Although a preliminary magnetic measurement of the PR interval's MF had been reported in 1978 (116), high-resolution (HR) MCG recordings of the PR segment were carried out independently both with unshielded MCG and in MSR since the early 1980s (27, 33, 117, 118). The physiological interpretation of HR MCG waveforms of the PR interval was controversial (34, 119–122) until the nature of the so-called “ramp-like” pattern recorded during the PR interval (Figure 3A) was definitely clarified within the development of software for automatic MF mapping and interactive subtraction. In fact, by subtracting the atrial repolarization field component from the whole PR interval's MF, a weaker remaining MF generated by sources moving from the AV junction downward along the interventricular septum was identified, consistent with the activation of the His–Purkinje System (123), coherently with the output of a more advanced mathematical model of the normal AV conduction pathways (124–126) and later by simultaneous MCG and invasive His bundle electrogram recording (127) and by direct comparison of atrial magnetic repolarization field distribution and simultaneous atrial monophasic action potential recording (128) (Figure 3B). Further results on MCG recording of His bundle activity were subsequently reported also by other studies conducted in MSRs (129–131).

### 2.1. From waveform analysis to cardiac magnetic field mapping and 3D magnetic source localization

While until the beginning of the 1980s MCG research focused on waveform analysis by comparing it with ECG, one of the most appealing potential features of MCG mapping was the ability to provide, at least in theory, accurate non-invasive 3D localization of intracardiac sources through the inverse problem solution with the relatively simple equivalent current dipole (ECD) and effective magnetic dipole (EMD) models (132, 133), or more advanced mathematical and regularization methods (134), at that time with better accuracy compared with body surface potential mapping (135, 136). The first attempts for MCG localization of the human His bundle (34), of the accessory pathways in the Wolff–Parkinson–White syndrome (35), and of supraventricular (137) and ventricular arrhythmias (138, 139) were initially validated by off-line comparison with the fluoroscopic position of intracardiac catheters recording the His’ and Kent bundles’ electrograms and ventricular fractionated activity, respectively (80, 123, 140–143).

Meanwhile, experimental validation of MCG 3D localization accuracy of intracardiac sources had been also preliminarily provided with specially constructed amagnetic catheters (ACs) generating current dipoles of variable geometry and intensity in a simple tank phantom filled with saline at first and then during the actual electrophysiological study in patients (144, 145). With sequential single-channel MCG mapping in Rome's unshielded hospital setting, average MCG 3D localization uncertainty of dipolar sources was in the order of about 12 mm in patients and about 5 mm in the phantom, good enough results to generate the original and patented concept to combine contactless MCG mapping with specifically designed ACs for minimally invasive (single-catheter) interventional electrophysiology study of arrhythmogenic substrates and for the magnetic guidance of endomyocardial biopsy and ablation (146–149).

Such promising results obtained with single-channel sequential MCG mapping were substantially confirmed by preliminary clinical findings obtained with a novel multichannel MCG system (KRENIKON, Siemens GMBH), which were presented at the workshop organized by the European Concerted Action (COMAC-BME) on Biomagnetism (Rome, December 1990) (150, 151). That multichannel MCG system provided accurate localization of the arrhythmogenic substrates of patients with the Wolff–Parkinson–White syndrome and with ventricular tachycardia validated with successful catheter ablation (152). With the same shielded multichannel mapping system, also the localization accuracy of pacing catheters was lately confirmed (48, 153).

All COMAC-BME's reported data reinforced the evidence that the localization of cardiac arrhythmias could be a relevant application of MCG with a good chance for further development. However, the consensus concluded that simultaneous multichannel MCG mapping was a mandatory requirement for clinical application.

**Figure 4:**
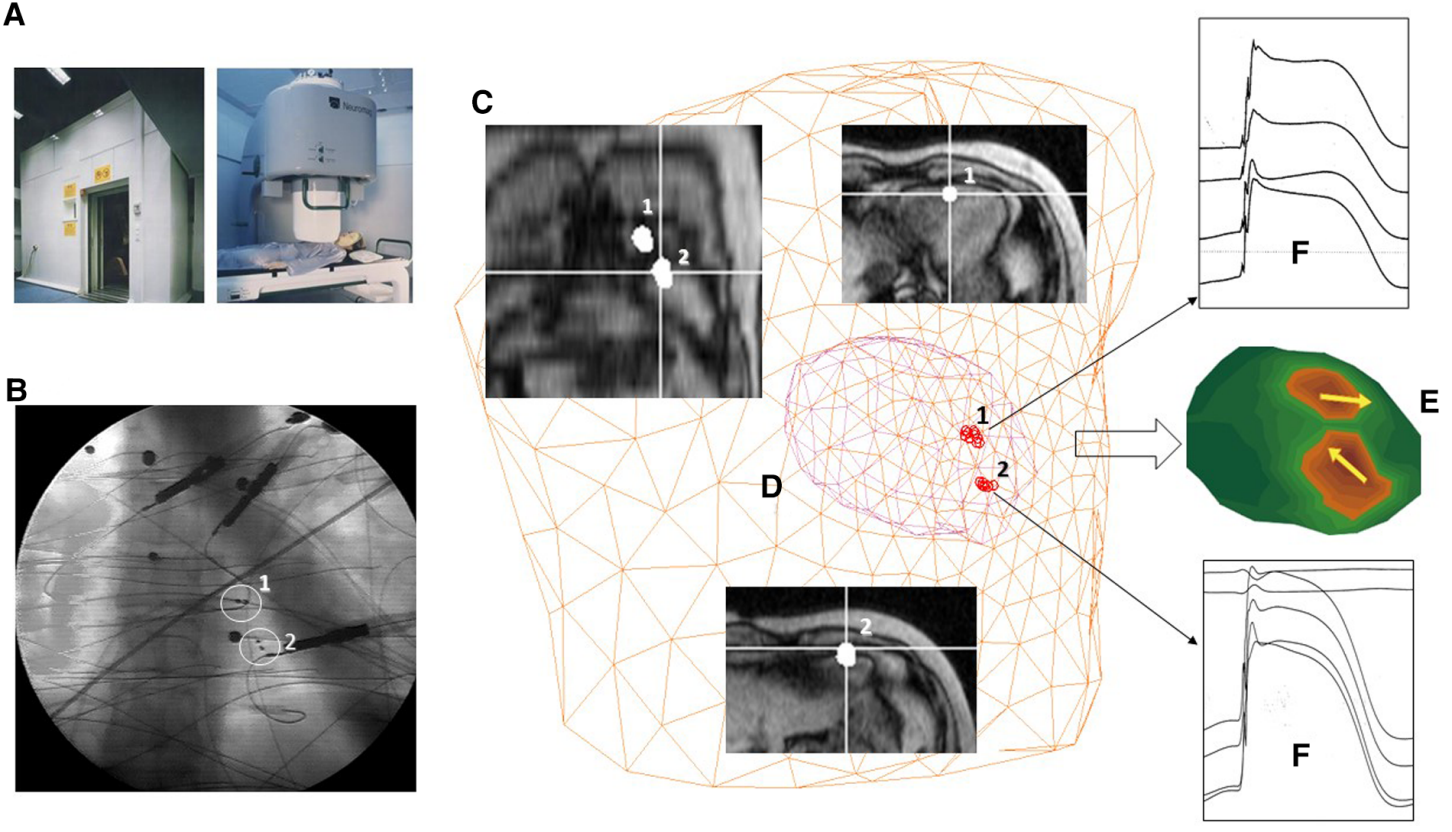
Example of multimodal 3D EAI based on MSI validated in the Helsinki University Central Hospital's BioMag Laboratory. (**A**) The MSR. (**B**) Fluoroscopic imaging of two amagnetic catheters (ACs). (**C**) MRI imaging. (**D**) 3D localization of the distal end of the ACs with the MCG ECD inverse solution. (**E**) 3D current density imaging of two 10 µA current dipoles generated by the ACs. (**F**) Multiple monophasic action potential recordings from the ACs.

A few years later, in the framework of the BIRCH-large-scale facility in a biomagnetism program selected by the FP3-HCM (Human Capital and Mobility) program (154), a research project to validate the accuracy of the improved amagnetic catheter technique for magnetically guided interventional electrophysiology and monophasic action potential recording was carried out with the high-performance Neuromag multichannel MCG system, installed in the MSR of the Helsinki University Central Hospital's BioMag Laboratory (Figure 4). For AC's dipolar sources placed within 10 cm from the sensors’ plane, the MCG 3D localization accuracy was optimal in a realistic torso phantom (average: 2 ± 0.7 mm SD) (155), as well as in patients (average 4 ± 2,7 mm SD), and about twice as better than that obtainable with simultaneous body surface potential mapping (155–157). Such results definitely validated the reliability of the multipurpose amagnetic catheter to generate reproducible artificial intracardiac sources to test the MCG 3D localization accuracy, providing also evidence that MCG could be used for non-fluoroscopic imaging (156, 157) to guide specifically designed ACs for the simultaneous high-resolution recording of multiple monophasic action potentials right into focal arrhythmogenic substrates, preliminary localized non-invasively with the same MCG system in ambulatory patients (158, 159). Moreover, the data acquired with the ACs’ technique were useful to evaluate the efficacy of different regularization methods for epicardial minimum norm estimates, to quantify the effects of geometric and topologic differences in boundary element models on magnetocardiographic localization accuracy of cardiac focal sources, and to validate the accuracy of equivalent current density reconstruction from MCG inverse solution in terms of the lead field and appropriate regularization techniques to stabilize the solution (160–163). Current density imaging was clinically applied to localize exercise-induced myocardial ischemia and focal arrhythmogenic substrates after myocardial infarction (55, 164–166).

At that time, compared with other invasive navigation systems (167–170) and non-invasive body surface potential mapping (171, 172), the novel cardiac magnetic source imaging (MSI) concept already had the unique capability to provide the same contactless and radiation-free instrumentation, accurate real-time integration of non-invasive preoperative 3D imaging of the arrhythmogenic substrates, and intraoperative electrophysiological single amagnetic catheter (128, 173).

Interestingly, in 2015, the higher localization accuracy of magnetic technology was also confirmed many years later by a phantom study carried out to compare the spatial localization reproducibility and catheters’ visual accuracy of two modern sensor-based electroanatomic navigation technologies (174).

### 2.2. From single-channel MCG mapping to multichannel MCG imaging in unshielded hospital settings

Although high-performance multichannel systems and the progressive development of mathematical algorithms combining patients’ cardiac 3D models obtained from MRI or CT scans improved the validation of cardiac MSI, such expensive gold standards with heavy EMS were available only to a limited number of clinicians. Among them, only a few, including our group in Rome, were foreseeing MCG as a novel tool to be introduced within current clinical practice at scale, by developing user-friendly multichannel medical devices working routinely in unshielded hospital cardiology ambulatories and electrophysiology labs. In our hands, it became possible after receiving a grant from the Italian National Ministry of Research to co-finance a joint research project with the newborn CardioMag Imaging Inc. (*CMI, Schenectady, United States*).

A more detailed description of the Catholic University's “Biomagnetism and Clinical Physiology International Center) (BACPIC)” clinical setup and investigational protocols can be found in the literature. Briefly, after installing in our unshielded laboratory for cardiac interventional electrophysiology the first CMI nine-channel MCG prototype (Figure 2B), whose reliability was validated for about 1 year (175), the first (and for a long time unique) unshielded 36-channel MCG (CMI 3619a) system became operational in January 2002 (Figure 2C), allowing since then ambulatory MCG assessment of a large number of patients, with immediate diagnostic-support feedback (67). With the latter device peak-to-peak background noise was 7–30 picotesla (pT) (raw signals) and 1–2 pT, after adaptive filtering of 50 Hz. After signal averaging, the sensitivity was 20–40 fT/√Hz above 1 Hz. The inverse solution of cardiac MF and the analysis of the EMD dynamics were highly reproducible (ICC: >0.7) in localizing cardiac sources. Quasi-real-time (90 s for mapping and less than 2 min for analysis) multimodal imaging of the cardiac MF dynamics (Figure 2D) became possible even during interventional electrophysiology (128, 176, 177). Ambulatory MCG study became a routine ambulatory procedure to study cardiac patients, including those with arrhythmias, to improve the non-invasive mechanistic diagnostic accuracy provided by electrocardiographic methods (67, 102). The multimodal integration of MCG localization results within a 3D model of cardiac anatomy, reconstructed from orthogonal fluoroscopic images (178) and/or from 3D rendering of cardiac MRI or CT scans, provided accurate pre-interventional localization of focal arrhythmogenic substrates, useful to guide catheter ablation. Additional validation of unshielded MCG was also provided by collaborating with other authors who had developed advanced software tools for 3D EAI of arrhythmogenic substrates (179–182), who independently elaborated some MCG files of our patients with atrial flutter or fibrillation, with reproducible results (183).

**Figure 5:**
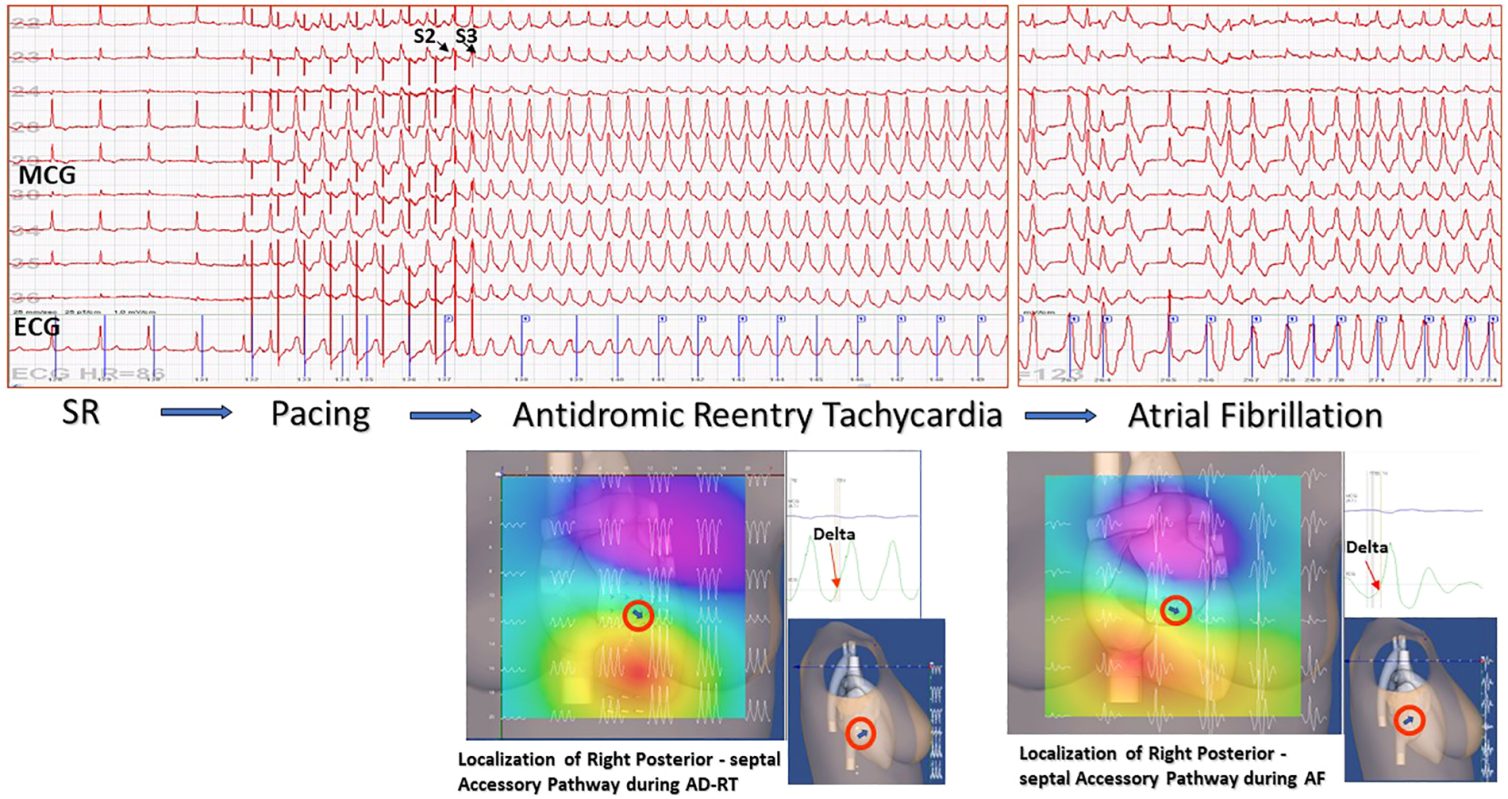
Example of non-invasive MCG localization of a right posterior-septal Kent bundle during sustained antidromic reentry tachycardia induced by transesophageal atrial pacing and spontaneous desynchronization in atrial fibrillation. The accessory pathway localization was confirmed by successful RF ablation.

Although all possible diagnostic applications of unshielded MCG, including the first multichannel mapping and source localization of the fetal heart, were explored in our center during the last two decades (68, 184–191), our main research focus has been to develop multimodal MSI-based non-invasive 3D EAI, during sinus rhythm and sustained arrhythmias, spontaneous or induced with transesophageal atrial pacing (137, 173) (Figure 5), to reduce the need for invasive electrophysiology (192, 193) and to minimize it, when eventually strongly indicated or unavoidable (128, 176, 194).

**Figure 6:**
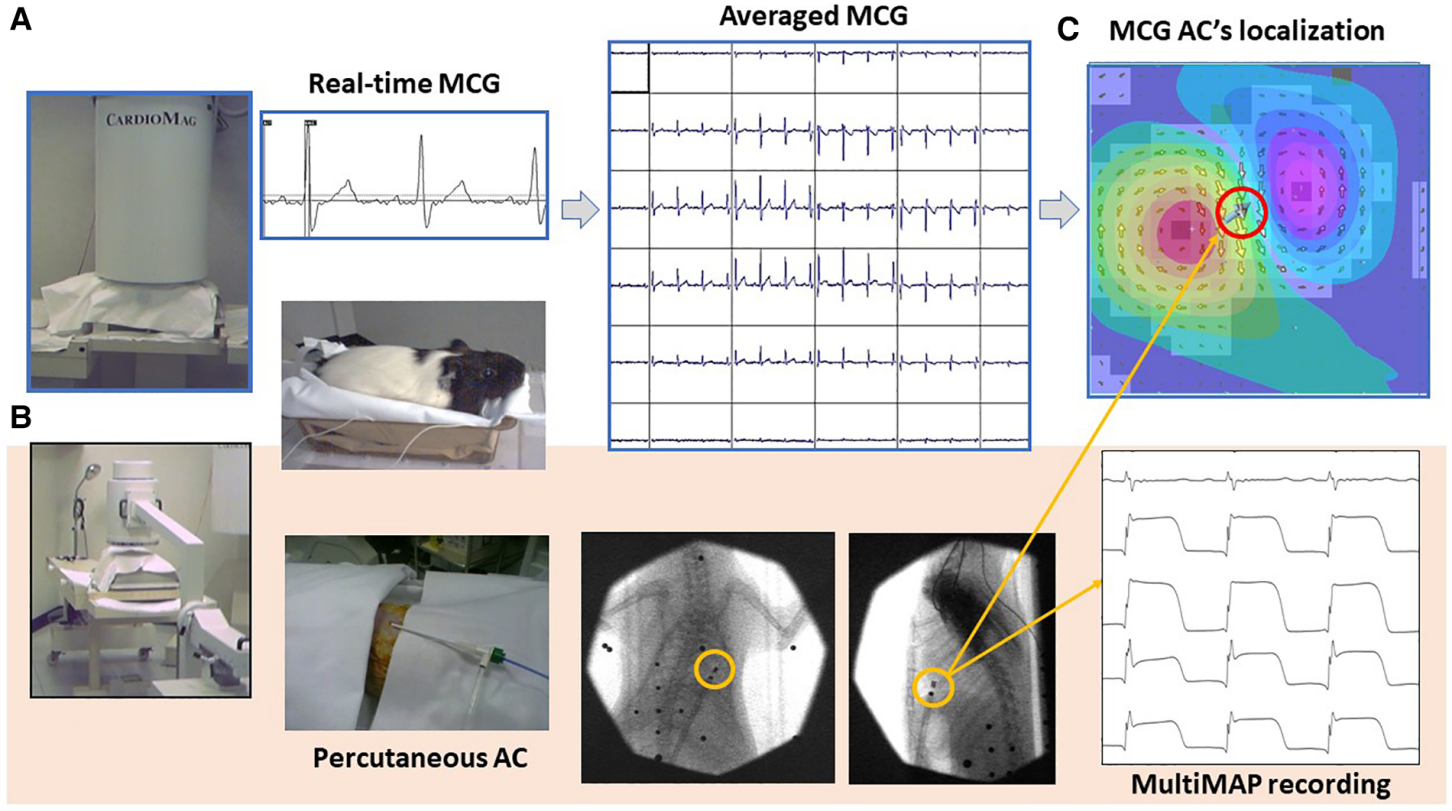
Typical experimental setup used for the MCG study of small animals with the CMI 3649a system. (**A**) Non-invasive contactless MCG recording and source imaging. (**B**) Procedure for simultaneous minimally invasive electrophysiological study with a single AC for multiple monophasic action potential recording from the epicardial surface, localizable with the MCG mapping and EMD inverse solution [red-circled solid arrow in (**C**)].

Last but not least, while aiming at further validating the accuracy of 3D EAI of the same unshielded CMI 3619a installation currently used for clinical MCG, mapping the cardiac MF of small animals (195) has been also performed, demonstrating that unshielded MCG is also feasible, reproducible, and reliable for the non-invasive contactless electrophysiological study of animal models, even with the integration of minimally invasive epicardial monophasic action potential recordings without animals’ sacrifice (Figure 6) (84, 196–200). The interest for MCG in animals’ experimental models is progressively increasing, and its application for the electrophysiological study of transgenic models of cardiomyopathy, experimental myocardial injury, and regulatory pharmacological preclinical evaluation has been confirmed by other authors, working in MSR with cryogenic instrumentations (201–206), with atomic optically pumped magnetometers (OPMs) (85, 89, 207), with nitrogen-vacancy (NV) diamond magnetometers (208), and with high-resolution fluxgate (209). An attempt for unshielded MCG recording in cattle with an OPM gradiometer system has also been reported (210).

During the same years, several other authors were working in clinical environments with smaller devices (in general up to nine channels) for sequential unshielded MCG mapping, providing additional evidence that the unshielded choice was reliable for the clinical application of MCG at scale. Among them, the CMI nine-channel prototype was the first to get an FDA approval for the measurement of human cardiac MF and was used for the first MCG multicenter clinical trial in the United States and in Europe as well as installed in a cardiology department in China. Most of the current lines of research aimed to electively investigate the predictive accuracy of MCG for the detection of myocardial ischemia (71, 72, 74, 76–78, 211–216) also during effort test (217) and the emergency triage of patients with chest pain (87, 88). Interesting data were also obtained in patients with non-ischemic arrhythmogenic cardiomyopathy (96, 138, 218, 219), confirmed also by more recent multichannel MCG studies in MSRs (220, 221). Further experimental (222) and clinical research has confirmed the reliability of unshielded MCG mapping as a unique method for the contactless ambulatory study of myocardial ischemia, for fetal MCG (223, 224), for risk assessment, and for follow-up of asymptomatic Brugada patients (225) to identify patients with complex ventricular preexcitation (226).

Parallel research conducted in MSRs has confirmed that MCG was an innovative and reliable tool for various clinical applications (88, 105, 212, 227) including but not limited to non-invasive 3D EAI but reaching routine use for diagnostic and prognostic-supported purposes in a few major hospitals only (105, 228–236). However, in spite of such evidence, MCG development has progressed slowly in the first decade of the year 2000, and, although with some exceptions, it is “de facto” still constrained to the research setting. On the contrary, after approximately 40 years after its birth (237, 238), following the roadmap suggested by MCG research (79), BSPM has nowadays got a real clinical breakthrough as a method for non-invasive 3D EAI, with the competitive concept of “ECG imaging” (ECGi) (239–241), a technology that has rapidly evolved in clinically available devices (242), increasingly used for non-invasive pre-interventional assessment of patients undergoing catheter ablation procedures (243, 244).

## 3. Unshielded MCG: the present

While clinical studies, which was carried out in specialized centers with shielded and unshielded SQUID-based MCG systems, enhanced the evidence for MCG 3D electroanatomical localization accuracy and for risk assessment of arrhythmogenic mechanisms/substrates (82, 83, 90, 226, 245–251), a renewed interest for biomagnetic sensor technology has raised again not only at the academic but also at the industrial level (252, 253). In fact, apart from the traditionally available multichannel cryogenic MCG systems working in MSRs (246, 254–259) and a more downscaled price for sequential unshielded MCG mapping (77, 256–259), three younger companies have manufactured innovative multichannel MCG devices, based on different sensor technologies, all of them specifically designed for clinical application at minimized running costs and with optimized operational simplicity. All of them have gathered regulatory clearance for the recording of human cardiac magnetic fields. Two operate in unshielded environments, while the third one still needs performant EMS, based on optical magnetometry.

### 3.1. A cryogenic system without the need for liquid helium transfer

**Figure 7:**
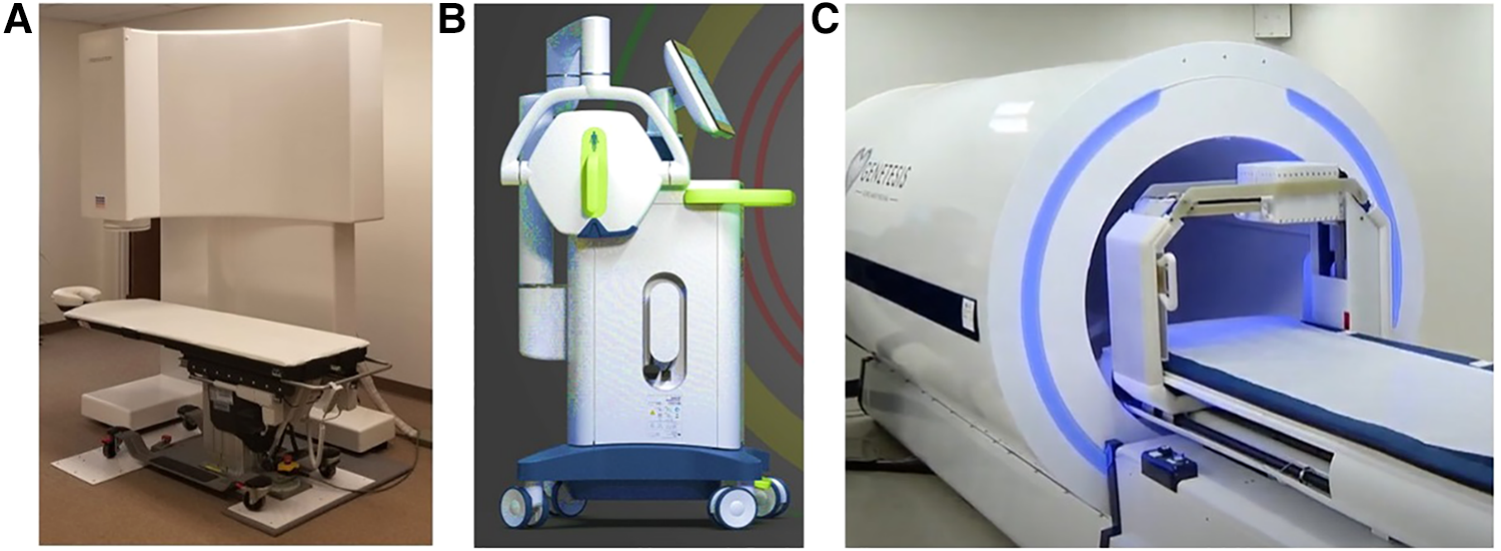
Examples of most recent novel MCG devices, two operating unshielded: (**A**) the cryogenic Avalon-H90. (**B**) The portable VitalScan/Corsens and one still requiring local EMS provided by a more compact cylindric MSR. (**C**) The CardioFlux, featuring zero-field OPMs.

The Avalon-H90 (Mesuron LLC, United States) features 67 measuring points of SQUID sensor array arranged in a way that the X, Y, and Z components of the cardiac MF can be simultaneously recorded at each point, thus providing MCG 3D imaging of cardiac electrophysiological events (Figure 7A). Although cryogenic, the Avalon-H90 using an integrated cryocooler is kept at the required low temperature without needing the weekly refill of liquid helium, thus avoiding the huge and increasing expenses of helium consumption. Furthermore, due to its inherent synchronization over the whole magnetic map, no ECG recording is needed either; thus, the exam is completely contactless. The system is proposed as reliable in regular unshielded hospital rooms, also in close proximity to other medical apparatus and electronic equipment, if they are located at a distance of at least 3 m from the sensor array. This completely innovative 3D vector MCG system design seems to fulfill also the functional requirements for non-fluoroscopic imaging to guide minimally invasive (e.g., the single-catheter electrophysiological study of MCG-localized arrhythmogenic substrates) (79, 147, 159). However, to the best of our knowledge, at present, the system's proprietary software is mostly addressed to multidimensional analysis of ventricular repolarization dynamics and to detect abnormalities due to ischemic and non-ischemic cardiomyopathies. The Avalon-H90 is under clinical evaluation for the triage of chest pain patients in the emergency department (ED).

### 3.2. A truly portable compact multichannel MCG device

An interesting innovative non-cryogenic alternative for unshielded multichannel MCG mapping was originally developed at the University of Leeds and could be ideal for ambulatory clinical use and in the ED, because of its easier portability to the patient's bedside (260). Designed with the aim to produce a clinically desired, feasible (261), and inexpensive device that could be rapidly deployed in any noisy unshielded ward environment (102, 261), it features novel compact mini-induction coil magnetometers assembled in a hexagonal 19-sensors array to detect MCG from a measurement surface of about 25 cm × 25 cm. A first pilot clinical study (protocol NCT02359773, on ClinicalTrials.gov) had shown that the device provided high sensitivity (95.4%) and a negative predictive value (NPV) (97.7%) for the rule-out of healthy subjects and of patients whose chest pain was non-ischemic from those with ischemic heart disease (262). However, a subsequent multicenter prospective cohort study evaluating the diagnostic accuracy of the MCG in adults with a suspected acute coronary syndrome (ACS) was conducted with their first generation of the derived industrial product (VitalScan/Corsens©, Creavo Medical Technologies, Coventry, United Kingdom) (Figure 7B) and concluded that, at least in 2020, the VitalScan did not yet meet the level of accuracy required to confidently rule out ACS in the ED clinical practice (263). More recently, another study carried out with a new 37-channel (19 active sensors and 18 for noise reduction) prototype by Leeds University suggests that, with appropriate modeling, 5 of 38 magnetic QRS parameters could be used to provide a MCG estimate of left ventricular ejection fraction (264). Although very appealing for its portability, user-friendly flexibility, and relatively lower cost, the potential of the device for 3D EAI has not been reported so far. However, new information about its reliability for arrhythmogenic risk assessment is expected from the results of the ongoing prospective MAGNETO-SCD study (265, 266).

Further preliminary research using a novel miniature induction coil array and digital signal processing algorithms to record unshielded MCG from a “simulated heart” (267) and to calculate heart rate variability under cognitive workload in healthy volunteers (268) has been recently reported. However, in the latter study, MCG signals were very noisy, and the QRS peaks identifiable in only 11 out of 13 participants, something that might still be a limitation for clinical use.

### 3.3. A still shielded but more compact and non-cryogenic (OPM-based) multichannel MCG system for emergency departments

Another non-cryogenic alternative to SQUID-based MCG systems is the use of OPMs (269). A theoretical overview of the physics background of OPMs is beyond the scope of this paper but can be found in an excellent recent paper (270). Briefly, OPMs (as MRI) are based on the manipulation of a property that underlies a particle's magnetic moment known as “spin” and its response to magnetic fields. Around the early 1960s, it was shown that optical pumping [i.e., the use of a (laser) light to induce absorption or emission of energy by a material sample] could be used for inducing a magnetically sensitive state in an atomic system and therefore allow for the measurement of even weak magnetic fields. After approximately 40 years, OPM technology has reached femtotesla sensitivity with the advantage to work at room temperature (RT) (271, 272) and can be placed closer to the body surface, thus recording MCG signals of higher amplitude compared with those reaching the cryogenic sensors, unavoidably more distant due to the Dewar's wall thickness. More recently, OPMs have improved the level of miniaturization making them even wearable, opening additional avenues for a more flexible use in multiple clinical applications (270, 273, 274), despite still requiring heavy EMS.

**Figure 8:**
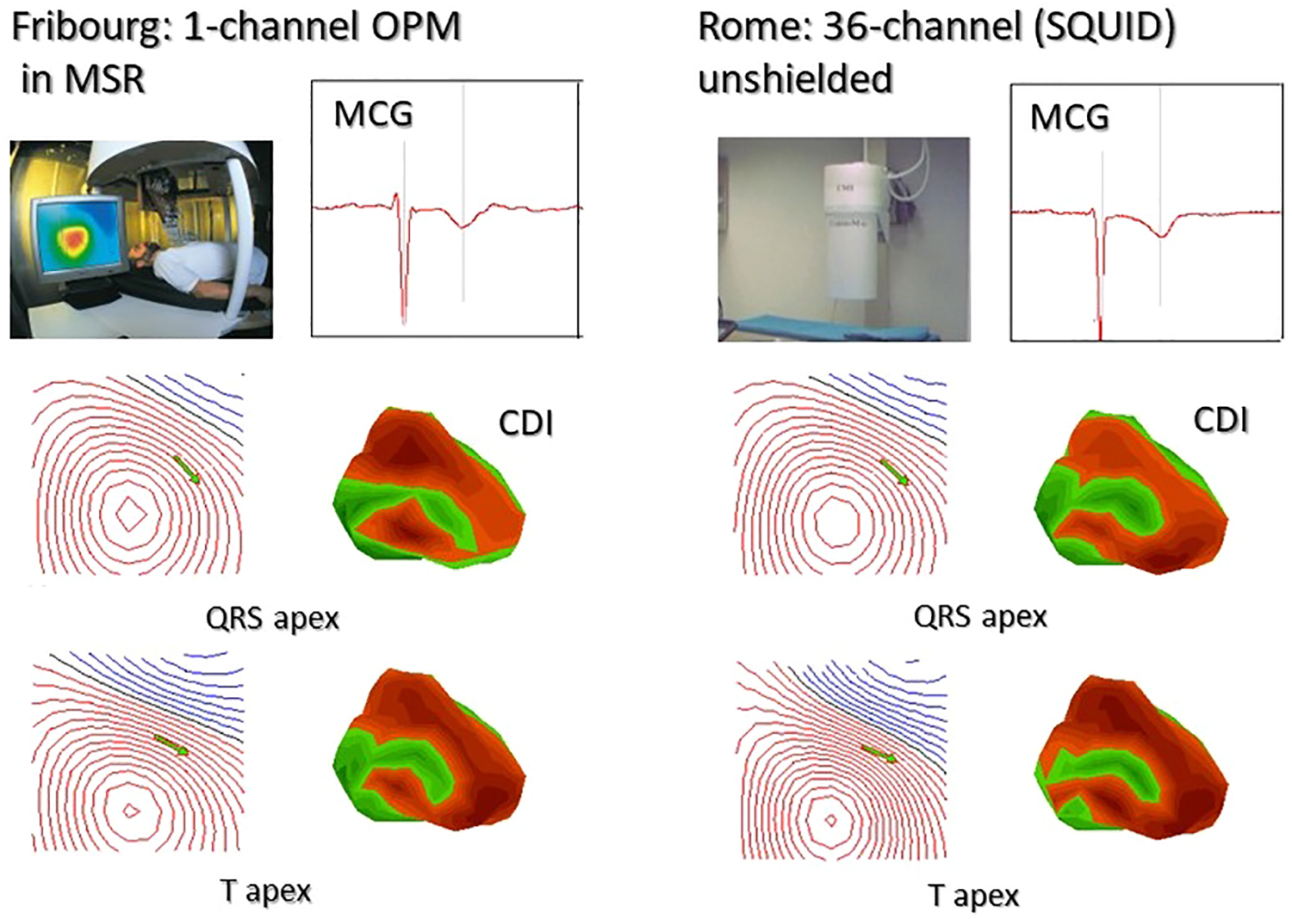
First reported comparison between sequential 36-point MCG recording with a single-channel OPM developed at the University of Freiburg (Swiss) and 36-channel MCG mapping at the Catholic University Hospital in Rome. All data were converted to the same format and analyzed with the Neuromag MCG software. The identity of MCG waveforms, of the MF distribution, of the related source localization (green arrows onto the MF maps), and of the 3D current density imaging (CDI), calculated at the apex of the QRS and of the T waves, is immediately evident [modified from data in (275)].

The first comparison between 3D localization accuracy of cardiac sources obtained with unshielded multichannel SQUID-based and shielded single-channel OPM MCG mapping of two normal subjects was reported at the Fourth International Conference “Noninvasive Functional Source Imaging (NFSI)” held in Chieti in 2003 (275) (Figure 8). The coincidence of the results was impressive, and the authors concluded “… we believe that, although at the moment still confined in a shielded room, OPMs have the potential to compete in a near future with cryogenic sensor, especially taking into account that OPM are potentially one order of magnitude less expensive than SQUID sensors and practically maintenance- and cost-free.”

Fourteen years later, in 2017, two Genetesis researchers visited the BACPIC within a research agreement with the Catholic University, and (first generation) QuSpin zero-field OPM sensors (QZFM) were tested in the BACPIC's unshielded biomagnetic catheterization laboratory, as the first step to develop an innovative OPM-based device for MCG 3D EAI of arrhythmias, featuring a similar 36-sensors grid geometry, data acquisition protocols, and analytic approach to facilitate the planned validation by comparison with BACPIC's CMI 3619a gold standard system (67, 87). Using zero-field OPM sensors, the CardioFlux system (Genetesis, Inc. Mason, United States) needs performant EMS, which is obtained by sliding the patients into a cylindrical MSR during the time required for data acquisition (Figure 7C). Since the CardioFlux prototype was assessed in clinical trials designed to evaluate patients presenting in ED with chest pain and suspected ischemic heart disease (276), the joint project was cancelled. The QuSpin zero-field OPMs were also more recently used to develop a wearable MCG mapping system (277). Although still needing EMS, such recent developments have provided a clear demonstration that the OPM technology is ready to compete with cryogenic sensors in terms of MCG sensitivity with bandwidth appropriate for clinical purpose. However, for a widespread adoption at scale, a “stepping out” from shielding is very much required.

## 4. Unshielded MCG: the future

### 4.1. Innovative OPMs for unshielded magnetocardiography

**Figure 9:**
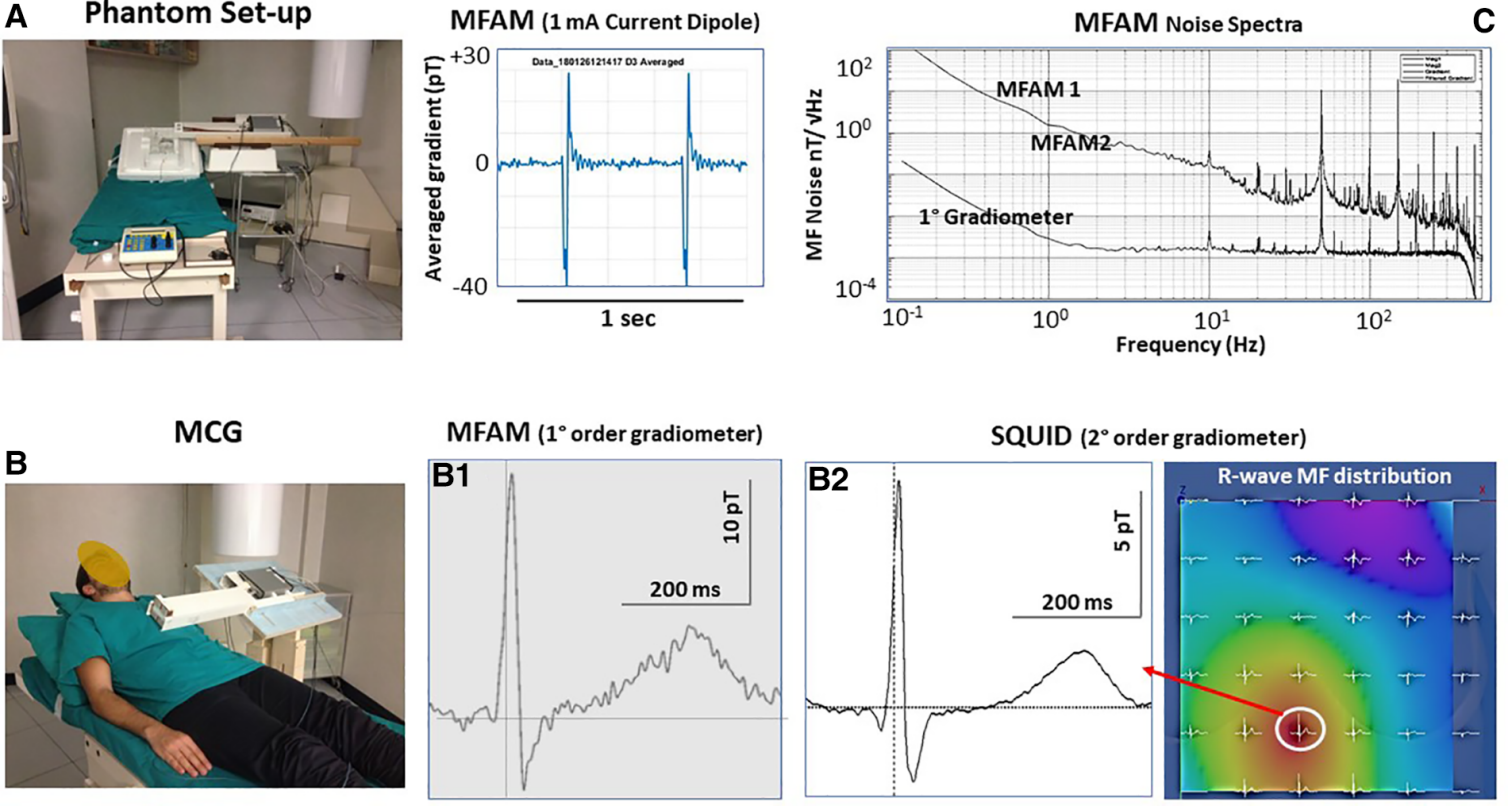
First reported test of a dual-channel scalar field OPMs (geometrics MFAM) arranged as a first-order gradiometer (baseline 5 cm) in the Catholic University Hospital's unshielded BACPIC: (**A**) Preliminary phantom measurement of a 1 mA current dipole generated by the amagnetic catheter. (**B**) Comparison between MCG recording and CMI 36-channel MCG mapping (R-wave MF distribution). The similarity of MCG waveforms recorded with the MFAM (**B1**) and with the SQUID (**B2**) at the same grid position (white circle onto the MF map) is evident. The amplitude of the MCG signal recorded with the MFAM is higher because the OPMs can be placed closer to the chest. (**C**) Noise spectra of each MFAM and of the first-order gradiometer.

Since the zero-field OPMs’ optimal sensitivity cannot be suitable for unshielded MCG at patients’ bedside in noisy unshielded hospital wards, an obvious alternative was to explore if the sensitivity achievable with scalar OPMs arranged in a gradiometric configuration to improve their SNR was adequate for unshielded MCG. The first generation of Miniature Scalar Atomic Magnetometers (MFAM™, Geometrics Inc., United States) originally developed for geophysics, operating within the Earth's MF with sensitivity better than 2 pT/√Hz to approximately 400 Hz, was reported to detect the cardiac MF in an unshielded office environment. The MFAM (in a first-order gradiometric configuration) was successfully tested in the BACPIC's unshielded catheterization laboratory, first with the ACs’ technique to generate current dipoles of different geometry and intensity in a phantom (Figure 9A), thereafter by comparing MFAM-MCG with SQUID-MCG of the same healthy volunteers sequentially recorded in the same unshielded laboratory (Figure 9B). MFAM OPMs were stable enough to record an almost artifact-free MCG, with a SNR adequate for unshielded clinical evaluation of ventricular de/repolarization, but unfortunately not yet of atrial electrophysiology. The authors concluded that a better performance of total field OPMs was foreseen with development of more efficient gradiometer technology (278).

In the same year, two almost simultaneously published papers were confirming that vision (279, 280), suggesting new scenarios for the near-future diffusion of unshielded clinical MCG at scale. Zhang et al. (279) developed an all-optical intrinsic scalar magnetic gradiometer composed of two miniaturized cesium vapor cells (inner dimension of 5 mm^3^ × 5 mm^3^ × 5 mm^3^) separated by a baseline of 5 cm and driven by one vertical-cavity surface-emitting laser and demonstrated a gradiometer output noise density of less than 90 fT/√Hz, which is equivalent to 18 fT/cm/√Hz sensitivity in the gradient measurement for a baseline of 5 cm. A better sensitivity (16 fT/cm/Hz^1/2^), good enough to detect biomagnetic signals generated from the human brain and heart in Earth's ambient environment, was reached by Limes et al. (280), with a 3 cm baseline gradiometer based on microfabricated OPMs using two ^87^Rb vapor cells (dimension 8 mm^3^ × 8 mm^3^ × 12.5 mm^3^), advanced thermal insulation, custom electronics, and compact laser within the sensor head, operated by the USB port of a laptop. Both technologies have also proven sufficiently sensitive for unshielded MEG and brain source localization using an array of scalar OPMs in the presence of a large background field (280–284).

The most recent innovative application of scalar OPMs comes from China, where two cesium OPMs based on the self-oscillating frequency tracking technique and a sensitivity of 140 fT/Hz^1/2^ have been successfully used for dynamic MCG recordings during real-life activities, including postural changes and exercise, and could be suitable long-term (Holter-like) MCG recordings (285), thus enhancing the potential diagnostic information of unshielded MCG.

The construction of more compact, portable multichannel instrumentations for MCG mapping and functional imaging in unshielded clinical environments based on such innovative OPMs is foreseen, although, to the best of our knowledge, such innovative technology is not yet commercially available. Another limitation for such development could be the present cost of scalar OPMs, since even commercially available low-sensitive (sensitivity: <0.2 pT/Hz^1/2^) sensors’ price is around $15 K per gradiometer unit, which is more than one order of magnitude higher than the present average cost of a SQUID. However, to decrease the cost by an order of magnitude, a multichannel OPM-based MCG system could be realized using a single large (or only a few) flat pancake rubidium vapor cell, broad pump and probe laser beams, and a multiple-channel photodiode array, as proven by the experimental 16-channel optically pumped magnetometer operating in the spin-exchange relaxation-free regime (SERF) developed by Kim et al. (286). Moreover, preliminary evidence has been provided that also SERF OPMs could be adapted in a gradiometric configuration potentially allowing unshielded MCG recordings (287, 288). At present optical magnetometry is the most advanced and promising alternative to cryogenic sensor technology. However, other alternatives are under development to reduce costs, some of them with appealing preliminary results.

### 4.2. NV diamond sensors

A future alternative to OPMs based on alkali-vapor cells could be using the negative NV center in diamond, which creates a spin system sensitive to an external MF that can be optically detected with a photodiode (289–293). These detectors can be made smaller than those using alkali metal vapors but at the moment are less sensitive and still require EMS. Since the best reported diamond magnetometers’ sensitivity is between 12 and 50 pT/Hz^1/2^, they are suitable for experimental recording of magnetic fields from isolated nerve or muscle neuronal action potentials isolated nerve or muscle tissues (294) or even for invasive, close-proximity, high-resolution MCG of living rats (208), but do not have sufficient sensitivity to compete with SQUIDs or OPMs for MCG clinical applications. However, future technological developments could change the current scenario. In fact, recently reported optimization of quantum NV diamond magnetometers applied for magnetoneurography and magnetomyography applications demonstrates the feasibility of NV sensor gradiometers and suggests their potential to be used without EMS with a sub-picotesla sensitivity (295).

### 4.3. Other magnetic sensor technologies

To develop low-cost devices for multichannel MCG recording in the picotesla range, different kinds of alternative sensors have been tested.

An Ultra-Sensitive Vector Magnetometer based on low-cost fluxgate with a noise level of less than 100 fT/Hz^1/2^ was reliable in mapping the three components of cardiac MF by direct measurement without additional calculations but in a MSR (296). However, no data are available about the performance of that technology in unshielded environment. Instead, real-time beat-to-beat MCG recording of ventricular activity was achieved in an unshielded environment with a novel magnetic induction (MI) sensor gradiometer featuring a noise level of lower than 1 pT/Hz^1/2^ at room temperature. After averaging 20 cycles, also the magnetic P wave was detected (297).

With a 30-channel RT magnetoresistive (MR) sensor array (TDK Corporation, Tokyo, Japan), the MCG P, QRS, and T waves were detectable by signals averaging 250–300 beats and validated by comparison with SQUID-MCG of the same subjects. All recordings were performed in a MSR (298). However, very promising results for the development of a low-cost device for unshielded multichannel MCG were recently achieved with an innovative microfabricated tunneling magnetoresistance (TMR) sensor technology featuring 14.1 pT_rms_ sensitivity in the frequency band between 0.2 and 100 Hz combined to digital decrease of the environmental and sensor noises (299). The MCG recorded in an unshielded office was good enough for the clinical study of ventricular de/repolarization, but not yet of atrial MF. Since the appropriate manufacturing reproducibility of RT sensors is not fully established yet, a method to calibrate the sensitivity of individual magnetic sensors before biomagnetic measurement has been also recently proposed (300).

Promising future perspectives for “*in vivo*” MCG measurements are also foreseen with low-cost miniaturized giant magnetoresistive (GMR) superconducting integrated sensors (301, 302) and with high-resolution magnetic sensors with giant magnetodielectric effect (MDE) at zero bias field (303) technologies, although their present sensitivity is much inferior to that of SQUID and OPMs. Finally, of interest could also be the superconductivity potential of graphene technology (304–306), yet to be validated for MCG clinical research applications.

### 4.4. Denoising methods

Independently from the kind of sensor technology chosen, another key point to make unshielded MCG reliable is the availability of efficient denoising methods with appropriate “preservation” of the cardiac magnetic frequency bandwidth containing important electrophysiological information of clinical interest.

Aside from traditionally used methods, such as signal averaging (307), digital filtering (308, 309), adaptive filtering, and independent component analysis (ICA) (310, 311) or real-time electronic noise subtraction (312), based on more advanced mathematical modeling and digital signal processing, several new solutions have been proposed, last but not least artificial intelligence (AI)-aided noise processing (313). The results of that study demonstrated that a better denoising performance of the proposed deep learning-based prediction model showed a larger noise reduction at low frequencies and lowered the 1/f knee frequency compared with the moving average filtering technique. The authors concluded that “with further adjustments to the preprocessing striding window-size in could be possible to tune out the low-frequency noise without affecting the MCG features.”

A noise reduction method based on the Ensemble Empirical Mode Decomposition (EEMD) technique improved (of approximately 18 dB) the SNR of MCG recorded with a four-channel low-Tc DC-SQUID system coupled to first-order gradiometers, in unshielded environment. The high correlation (r = 0.9) obtained between shielded MCG of healthy volunteers and unshielded MCG of the same subjects after EEMD denoising demonstrates that MCG of clinically acceptable quality can be recorded in unshielded environment even with first-order gradiometers if signals are processed with an efficient denoising method (314, 315). Further improvement could be expected with the improved variational mode decomposition (VMD) and interval thresholding (IT) method reported by Liao et al. in 2018 (316) and by introducing a system for active noise control (ANC) of environmental magnetic fields (317, 318).

## 5. Discussion

Magnetocardiography and in particular unshielded MCG are celebrating its 60th birthday, but is not yet a widely diffused and well-accepted clinical tool, although theory and experimental research suggest and confirm that it can provide additional diagnostic information not achievable with electrocardiography alone (1, 2, 4–6, 9, 16, 107).

Indeed, a large body of literature has provided evidence that MCG enhances the non-invasive diagnostic and prognostic capability in numerous clinical fields, such as but not limited to the earlier detection of (sometimes electrically silent) myocardial ischemia (59, 88, 90, 323, 319), the preventive arrhythmogenic risk assessment in adults (53, 225, 325–322) as well as in fetuses (83, 323, 324), and the diagnosis of inflammatory cardiomyopathy (40, 87, 218, 325) and of microvascular diseases (96, 326).

The usefulness of MCG for a better understanding of the arrhythmogenic mechanisms underlying the high-risk J syndromes (225, 327–336); the identification of atrial arrhythmogenic vulnerability (248), of left atrial dysfunction (330), and of the atrial propagation pathways (228); and the dominant frequencies (181, 183, 189, 232) in patients with atrial fibrillation, as well as providing accurate pre-interventional 3D localization of arrhythmogenic substrates (128, 193, 247, 249, 331), has been widely reported. However, the lack of a clinical breakthrough at scale is evident.

Trying to identify the possible reasons for such translational difficulty, already in 2005, we had suggested two major causes that (1) MCG technology was at that time still too sophisticated, expensive, and not available to the patient's bedside and (2) a sort of vicious circle, where the unavailability of MCG devices easily applicable to the clinical setting with the lack of a widespread knowledge and understanding of its potential, and some uncertainty on the results from clinical trial addressing some specific indications, enhanced the skepticism of both clinicians and investors with consequent persistent lack of sufficient resources to accelerate the research and development of novel and scalable clinical devices.

The “chimera” to solve the problem with highly reliable large-scale MCG systems providing the best signal quality in MSRs was predominant but contrasted with the “paradox” of trying to introduce new huge, highly expensive, and relatively complex-to-operate experimental technologies within the established electrocardiology world, where numerous standard solutions were already available. For decades, we had suggested and pursued a vision centered on the development of reliable, budget-priced, portable, and preferentially non-cryogenic, user-friendly devices for multimodal cardiomagnetic imaging, with software tools to simplify data fusion with other imaging techniques, as probably the most reasonable and effective solution to favor the diffusion of MCG at scale (102). However, the times and technology were not ready, in spite of evidence that, with appropriate adaptive solutions, multichannel MCG was feasible and reliable even in a clinical laboratory fully equipped for interventional cardiac electrophysiology (67).

Nowadays, the historical (and sometime hysterical) “to shield or not to shield” debate is going to become obsolete. In fact, from one side, improved shielding technologies provide flexible solutions, more easily adaptable to clinical environments and specific needs, at affordable costs. On the other hand, the new miniaturized non-cryogenic OPMs featuring a sensitivity comparable to the SQUID sensors aimed to be wearable for cardiomagnetism and neuromagnetism. Thus, using sensors which still need a MSR (e.g., the zero-field OPMs and the MR sensors), even those MCG devices could be used in time-sharing with MEG, within the same downscaled MSR, with sensitive reduction of the relative costs. Obviously, high-performance EMS still remains the preferred choice when the highest possible high resolution is needed, especially for experimental research.

Instead, as concerns most routine clinical MCG applications, there is an increasing evidence that progress in magnetic sensor technology combined with more advanced signal denoising methods is rapidly reaching the requirements for the near-future availability of several novel device variants for budget-price unshielded MCG, covering the needs of different deployment scenarios, from ED triage for suspected ACS (87, 259) to large-scale screenings of ambulatory patients (189) and to contactless monitoring during interventional cardiology (128) or badly burned patients and even foreseen for dynamic long-term assessment of cardiac MF dynamics (285).

The concept was clearly demonstrated by the excellent work by the Indira Gandhi Centre for Atomic Research's (IGCAR) researchers who, after EEMD processing, obtained a similar MCG signal quality with their DC-SQUID-based first gradiometer inside and outside the MSR (314); thus, we could anticipate that a similar result could at least be obtained with the most recent high-sensitivity scalar OPMs (279, 280, 282, 323, 319) and perhaps in the future even with zero-field SERF OPM gradiometers (325). However, since almost all those new OPMs were originally developed for MEG, their sensitivity and accuracy for deeper cardiac source localization need to be evaluated. Experimental use of much cheaper sensor would reduce the development and retailed costs of future devices for unshielded multichannel MCG. However, depending on technology, some of them still need EMS or at the moment have less sensitivity compared with SQUID and OPMs. Aside from hardware development, another limitation for MCG clinical adoption is the relative lack of software for MCG 3D EAI, at least non-inferior to that provided by recent ECGi. In fact, after its first appearance as a clinical tool (242), advanced software for ECGi and its integration with invasive 3D EAI has rapidly become available and is nowadays currently used for non-invasive study of arrhythmogenic electrophysiological mechanism and pre-interventional localization study of target substrates (243, 244, 320, 321), despite some pitfalls found in validation studies with the isolated and perfused pig heart model (322) and by comparison with invasive contact mapping (323). Might MCG 3D EAI be more competitive? For 3D localization accuracy of the arrhythmogenic site of origin, surely yes (181, 246, 247, 249). However, dedicated software packages for MCG endocardial and epicardial activation imaging and automatic integration with invasive 3D EAI are still lacking. With more adequate software tools, the advantages of 3D EAI based on unshielded MCG would be the speed and comfort of contactless mapping, to be inexpensive (no need of consumables, if performed with non-cryogenic devices) and better patient acceptance.

The development of wearable MCG devices may apparently seem in contrast with the MCG benefit of being a contactless method for easier and more comfortable cardiac mapping. Indeed, although a wearable device could theoretically favor a more realistic evaluation of cardiac MF dynamics (285), movement artifacts could markedly affect the reliability of such kind of measurements. However, an appropriately designed wearable mapping system (250) could be highly useful to improve MCG sensitivity and localization accuracy of weaker and deeper cardiac sources independently from their orientation, thus avoiding the need for simultaneous BSPM or favoring optimal integration of the two mapping methods for more accurate 3D/4D EAI (324).

Although the primary endpoints of the majority of MCG clinical trials have mostly focused on the demonstration of higher predictive accuracy of the method to diagnose or exclude ischemic heart disease (87, 88), at any possible stage from acute coronary syndromes to microvessel dysfunction, in this review, we have tried to summarize several relevant points demonstrating that the value of the information provided by cardiac MF recordings extends much beyond that clinical target. In fact, when the ECG and cardiac enzyme patterns cannot yet be diagnostic, the higher sensitivity of MCG in detecting myocardial ischemia at an early stage is only one of the advantages arising by the more comprehensive biomagnetic electrophysiological assessment, which has the potential to non-invasively detect electrogenic phenomena at cellular and even subcellular levels (86, 208). Moreover, as experimentally demonstrated by Cohen with DC-MCG measurements, it is possible to detect an ischemia-related diastolic injury current (106). Such “silent” abnormal electrotonic current flowing may usually arise at the border zone of an ischemic zone and can be arrhythmogenic if reaching the excitability threshold of the surrounding normal tissue (166). Unfortunately, although requiring EMS, DC-MCG is also clinically feasible (107) and with appropriate 3D electroanatomical integration with the structural and functional imaging provided by cardiac MR delayed enhancement imaging, it could be theoretically possible to detect and localize a potentially lethal injury current before it reaches the strength to generate a sustained VT/VF and sudden death in post-MI patients or in other kinds of advanced cardiomyopathies with extended pathological anisotropy.

Another example of unexplored potential information of MCG is suggested by a seminal experimental study of Benjamin Scherlag (the father of the method for clinical recording of the His bundle electrogram), who demonstrated that graded low-level EMFs applied to either the left or the right vagosympathetic trunks alter the sinus heart rate, the AV conduction, and the heart rhythm, facilitating the pacing inducibility of atrial fibrillation, in a canine model (325). Similar effects were observed when the EMF was applied with two larger Helmuth coils surrounding the whole chest and focused on the dog's heart. Interestingly, in a subsequent study, the same authors found that a pulsed EMF applied to the vagal trunks, or non-invasively across the dog's chest, can significantly reverse AF inducibility by inhibiting the neural activity of the atrial ganglionated plexus (326). Another elegant research has shown that non-invasive low-frequency electromagnetic stimulation of the left stellate ganglion (LSG) also reduces the occurrence of ventricular arrhythmia induced by acute myocardial infarction in a canine model (327). How this research connects to MCG? Indeed, it does. In fact, as pointed out by Wang et al., although many mechanisms, which might provide the basis for how the animals detect magnetic fields, have been proposed., the mode of transduction for the magnetic sense remains unknown. Following the same biomagnetic approach to study with MEG the effect of transcranial magnetic stimulation, MCG could be the right tool to explore and understand how noninvasive electromagnetic stimulation of cardiac autonomic innervation may affect cardiac electrophysiology, with proarrhythmic or antiarrhythmic effects (327).

Finally, since it is nowadays well known that the heart has its own “little brain” (328) and that the “heart-to-brain” afferences are at least functionally relevant as the “brain-to-heart” efferences, it can be hypothesized that the cardiac MF could be a third independent wireless “heart-to-brain” communication pathway, as preliminarily advanced by the HeartMath Institute's research (329). Simultaneous MCG and MEG mapping recording could shed some new light about the real possibility of contactless heart–brain synchronization. Practically impossible until now for the limitation related to the dimension and costs of cryogenic instrumentations, such research could become soon feasible with the novel OPM-based wearable MEG and MCG devices and in the near future even in unshielded environments (277, 279–281, 285, 336, 330).

## 6. Conclusion

Roth has concluded his interesting review of the first 60 years of biomagnetism by stating that it “remains a growing and developing field of study,” and wishing that “the next sixty years of biomagnetism might well be more momentous than the first sixty” (331).

As concerns the future of MCG, we are somehow more optimistic, because, as summarized here, decades of research have demonstrated that unshielded MCG is feasible and reliable providing the same information of clinical interest obtained with more expensive and bulky MCG systems working in MSR. Therefore, we feel confident that MCG is ready to reach its clinical breakthrough very soon.

Surely the future of MCG will continue in double parallel rails, shielded and unshielded, depending on local needs and finalities. However, whereas heavy EMS will remain a prerogative of highly specialized research centers, we definitely believe that only the development of reliable user-friendly unshielded devices will favor the widespread acceptance of MCG at the clinical level. Luckily, after many years of stagnation, present acceleration of sensor technology, and implementation of more efficient active shielding combined with AI-based signal denoising methods, we foresee that a larger choice of budget-price instrumentations for unshielded MCG mapping will be available in a relatively short time, allowing the diffusion of such novel medical devices at scale in hospitals and their clinical validation in the real world, initially as a routine add-on to standardized electrocardiography, but soon as a more rapid and efficient method for more comprehensive non-invasive electrophysiological evaluation, especially in centers where the high number of patients is to be screened. In fact, it takes less time for a standard 90-s (or even 5-min) MCG mapping than to perform a standard 12-lead ECG, especially in older patients with limited mobility.

As concerns the presently available new systems under the clinical testing, SQUID sensors are still the reference gold standard with the best sensitivity in unshielded environments; thus, a standalone novel multichannel vector mapping instrumentations (e.g., Figure 7A) with an efficient permanent cooling to get rid of liquid helium consumption may represent an intelligent more advanced cost-effective solution for multipurpose clinical applications of unshielded clinical MCG, including real-time 3D magnetic mapping and imaging during stress/pharmacological tests (225, 332–334), minimally invasive interventional procedures (128), and fetal MCG (185).

On the other hand, for large-scale routine ambulatory applications and to bring MCG to the patient's bedside, more compact movable devices fully reliable in unshielded hospital rooms are necessary (e.g., Figure 7B), obviously based on non-cryogenic sensor technologies, whose cost however might widely differ, depending on the type of sensors chosen as the front end.

Downscaled MSRs may be an alternative to profit of the higher sensitivity of zero-field OPMs even in noisy hospital environments (e.g., Figure 7C), if one accepts the limitations of partial visual control of the patients during data acquisition, the need for a wide space, and the problem of claustrophobia.

Apart from hardware developments, for clinical applications, looking at MCG as a powerful method for contactless and radiation-free 3D and potentially 4D functional imaging of cardiac electrophysiology (335), the development of more advanced software tools to merge MCG information in real time with other non-fluoroscopic (and/or nuclear medicine) imaging techniques (e.g., MRI and/or echocardiography (336) is absolutely a primary target to focus on. Multimodal 4D integration of magnetic current density imaging could become an unrivaled method to non-invasively investigate the dynamics of arrhythmogenic mechanisms occurring into the abnormal substrates due to ischemic and non-ischemic cardiomyopathies (including some channelopathies) (337). This would obviously allow more precise pre-interventional planning of the best interventional approach (e.g., endocardial vs. epicardial) for catheter ablation or for CRT or CCM treatments.

Last but not least, the standardization of MCG data acquisition, postprocessing, and analytic methods is required, providing shared and validated diagnostic criteria, independently of the hardware technology used in different devices. A tentative standardization of MCG was already defined by an “ad hoc committee” many years ago, during the NATO Conference on Biomagnetism (43) and the Fourth International Conference on Biomagnetism (338), held in Rome in 1982. However, even simple recommendations [e.g., the color code for MF interpretation, as specified in Figure 2, or the software tools to convert MCG data to a common format (339)], remained essentially unfollowed along decades of research, so that the results of MCG studies (and of clinical trials) so far carried out are only partially comparable and even less sharable in a common database.

## 7. Summary

Magnetocardiography is a multipurpose tool for contactless non-invasive functional imaging of cardiac electrophysiology, providing additional diagnostic information and more comprehensive electroanatomical imaging compared with electrocardiographic methods only. However, after more than 50 years of experimental and clinical research, MCG has not yet been accepted as a diagnostic method at scale.

In this review, relevant MCG milestones, achieved with cryogenic and non-cryogenic magnetic sensors operating in shielded and unshielded experimental and clinical setups, have been referenced and compared. Possible reasons for the still missing acceptability in the real clinical world have been discussed, based on over four decades of personal experience.

The unshielded approach has proven reliable to provide information of diagnostic interest equivalent to those obtained with more demanding systems needing heavy electromagnetic shielding. This suggests that the availability of next-generation unshielded devices will finally bring MCG to the patient's bedside, favoring its clinical application at scale.

The ongoing progress in magnetic sensors, active shielding, and denoising technology let us foresee the development of innovative solutions enhancing the reliability of unshielded MCG and the possibility to produce reliable and portable instrumentations, better tailored for specific clinical applications through optimal integration with other non-invasive imaging techniques and with electrocardiographic recordings when appropriate.

Finally, although the lack of standardization and the wide variability of MCG instrumentations, local experimental and clinical setups, and investigational protocols has made it difficult so far, a joint effort to centralize all potentially available MCG “big data,” converted into a common format with appropriate digital tools [e.g., (339)], could provide a valuable sharable database for wide retrospective studies demonstrating the real diagnostic potential of MCG based on statistically robust analyses. Such information would be extremely important to guide the development of novel MCG medical devices and the design of future more coordinated prospective multicenter clinical studies.

## References

[1] Plonsey R. Comparative capabilities of electrocardiography and magnetocardiography. Am J Cardiol. (1972) 29(5):735–6. 10.1016/0002-9149(72)90179-8 5021505

[2] Malmivuo J Plonsey R. Bioelectromagnetism—principles and applications of bioelectric and biomagnetic fields (1995). BioLabor Biofizikai és Laboratóriumi Szolg. Kft. Available at: www.biolabor.hu. (last accessed 02 July 2023)

[3] Grynszpan F Geselowitz DB. Model studies of the magnetocardiogram. Biophys J. (1973) 13(9):911–25. 10.1016/S0006-3495(73)86034-5 4733699 PMC1484372

[4] Wikswo JPJ Barach JP. Possible sources of new information in the magnetocardiogram. J Theor Biol. (1982) 95(4):721–9. 10.1016/0022-5193(82)90350-2 7109652

[5] Roth BJ Wikswo JP. Electrically silent magnetic fields. Biophys J. (1986) 50(4):739–45. 10.1016/S0006-3495(86)83513-5 3779008 PMC1329851

[6] Lant J Stroink G ten Voorde B Horacek BM Montague TJ. Complementary nature of electrocardiographic and magnetocardiographic data in patients with ischemic heart disease. J Electrocardiol. (1990) 23(4):315–22. 10.1016/0022-0736(90)90121-H 2254702

[7] Stroink G. Principles of cardiomagnetism. In: Williamson SJ Hoke M Stroink G Kotani M, editors. Advances in biomagnetism. Boston, MA: Springer US (1989). p. 47–56. Available at: 10.1007/978-1-4613-0581-1_4. (last accessed 02 July 2023)

[8] Stratbucker RA Hyde CM Wixson SE. The magnetocardiogram—a new approach to the fields surrounding the heart. IEEE Trans Biomed Eng. (1963) 10:145–9. 10.1109/tbmel.1963.4322823 14121114

[9] Baule GM McFee R. Detection of the magnetic field of the heart. Am Heart J. (1963) 66:95–6. 10.1016/0002-8703(63)90075-9 14045992

[10] Zimmerman JE Thiene P Harding JT. Design and operation of stable rf-biased superconducting point-contact quantum devices, and a note on the properties of perfectly clean metal contacts. J Appl Phys. (1970) 41(4):1572–80. 10.1063/1.1659074

[11] Cohen D. Shielded facility for low level magnetic measurements. J Appl Phys. (1967) 38:1295. 10.1063/1.1709590

[12] Cohen D. Magnetic fields around the torso: production by electrical activity of the human heart. Science. (1967) 156(3775):652–4. 10.1126/science.156.3775.652 6023659

[13] Cohen D Edelsack EA Zimmerman JE. Magnetocardiograms taken inside a shielded room with a superconducting point-contact magnetometer. Appl Phys Lett. (1970) 16(7):278–80. 10.1063/1.1653195

[14] Cohen D. Magnetoencephalography: detection of the brain's electrical activity with a super-conducting magnetometer. Science. (1972) 175(4022):664–6. 10.1126/science.175.4022.664 5009769

[15] Baule GM McFee R. The magnetic heart vector. Am Heart J. (1970) 79(2):223–36. 10.1016/0002-8703(70)90312-1 5410953

[16] Cohen D Kaufman LA. Magnetic determination of the relationship between the S-T segment shift and the injury current produced by coronary artery occlusion. Circ Res. (1975) 36(3):414–24. 10.1161/01.RES.36.3.414 1111998

[17] Cohen D Lepeschkin E Hosaka H Massell BF Myers G. Part I: abnormal patterns and physiological variations in magnetocardiograms. J Electrocardiol. (1976) 9(4):398–409. 10.1016/S0022-0736(76)80040-4 135816

[18] Denis B Machecourt J Favier C Martin-Noel P. Interpretation of the normal and pathological magnetocardiogram, based on 140 thoracic chestings (author's Transl). Ann Cardiol Angeiol. (1978) 27(1):81–6. 148865

[19] Geselowitz DB. Magnetocardiography: an overview. IEEE Trans Biomed Eng. (1979) 26(9):497–504. 10.1109/TBME.1979.326430 396218

[20] Cohen D Edelsack EA Zimmerman JE. Magnetocardiograms taken inside a shielded room with a superconducting point-contact magnetometer. Appl Phys Lett. (1970) 16:80–278. 10.1063/1.1653195

[21] Tumanovskiĭ MN Safonov ID Provotorov VM. Certain aspects of the clinical application of magnetocardiography. Kardiologiia. (1967) 7(4):70–5. 4233905

[22] Erné SN Hahlbohm H-D Lübbig H. editors. Biomagnetism: Proceedings. Third International Workshop, Berlin (West), May 1980. Berlin, New York: De Gruyter (1981). Available at: 10.1515/9783110863529. (last accessed 02 July 2023)

[23] Zimmerman JE Frederick NV. Miniature ultrasensitive superconducting magnetic gradiometer and its use in cardiography and other applications. Appl Phys Lett. (1971) 19(1):16–9. 10.1063/1.1653725

[24] Saarinen M Karp PJ Katila TE Siltanen P. The magnetocardiogram in cardiac disorders1. Cardiovasc Res. (1974) 8(6):820–34. 10.1093/cvr/8.6.820 4141924

[25] Saarinen M Siltanen P Karp PJ Katila TE. The normal magnetocardiogram: i morphology. Ann Clin Res. (1978) 10(Suppl 2):1–43. 677808

[26] Romani GL Williamson SJ Kaufman L. Biomagnetic instrumentation. Rev Sci Instrum. (1982) 53(12):1815–45. 10.1063/1.1136907 6760371

[27] Barbanera S Carelli P Fenici RR Leoni R Modena I Leoni R Use of a superconducting instrumentation for biomagnetic measurements performed in a hospital. IEEE Trans Magn. (1981) 17(1):849–52. 10.1109/TMAG.1981.1061052

[28] Nakaya Y. Magnetocardiography: a comparison with electrocardiography. J Cardiogr Suppl. (1984) (3):31–40. 6242156

[29] Nakaya Y Nomura M Fujino K Ishihara S Mori H. The T wave abnormality in the magnetocardiogram. Front Med Biol Eng. (1989) 1(3):183–92. Available at: http://www.ncbi.nlm.nih.gov/pubmed/2486759 (Accessed February 20, 2017). 2486759

[30] Vvedensky VL Naurzakov SP Ozhogin VI Shabanov SY. A portable biomagnetic measuring system. Nuovo Cim D. (1983) 2(2):224–30. 10.1007/BF02455926

[31] Stroink G. Principles of cardiomagnetism. In: Williamson SJ Hoke M Stroink G Kotani M, editors. Advances in biomagnetism. Boston, MA: Springer US (1989). p. 47–56.

[32] Katila T. 2 The beginning of biomagnetism and magnetoencephalography research in Finland in the 1970s. In: Papanicolaou AC Roberts TPL Wheless JW, editors. Fifty years of magnetoencephalography: beginnings, technical advances, and applications. Helsinki: Oxford University Press (2020). p. 8–21. Available at: 10.1093/oso/9780190935689.003.0002. (last accessed 02 July 2023)

[33] Fenici R Romani GL Barbanera S Zeppilli P Carelli P Modena I. High resolution magnetocardiography. Non-invasive investigation of the His-Purkinje system activity in man. G Ital Cardiol. (1980) 10(10):1366–70. 7239084

[34] Fenici RR Romani GL Leoni R. Magnetic measurements and modelling for the investigation of the human-heart conduction system. Nuovo Cim D. (1983) 2(2):280–90. 10.1007/BF02455931

[35] Fenici R Masselli M Lopez L Sabetta F. First simultaneous magnetocardiographic and invasive Kent bundle localization in man. New Trends Arrhythm. (1985) 1(3):455–60.

[36] Wikswo J Fairbank W. Application of superconducting magnetometers to the measurement of the vector magnetocardiogram. IEEE Trans Magn. (1977) 13(1):354–7. 10.1109/TMAG.1977.1059333

[37] Malmivuo JA Wikswo Jun JP Barry WH Harrison DC Fairbank WM. Consistent system of rectangular and spherical co-ordinates for electrocardiography and magnetocardiography. Med Biol Eng Comput. (1977) 15(4):413–5. 10.1007/BF02457995 197335

[38] Malmivuo J. On the detection of the magnetic heart vector—an application of the reciprocity theorem. [doctor of technology (PhD) thesis]. Acta Polytechnica Scandinavica, Electrical Engineering Series. Helsinki: Helsinki University of Technology (1976).

[39] Jensen O. *The 22nd international conference on biomagnetism (biomag)* (2022). Available at: https://biomag2020.org/wp-content/uploads/2022/08/Biomag-Conference-Brochure-POSTERS-v3_Spreads.pdf. (last accessed 02 July 2023)

[40] Heidecker B. Rediscovery of magnetocardiography for diagnostic screening and monitoring of treatment response in cardiology. Eur Heart J. (2023) 44(24):2140–2. 10.1093/eurheartj/ehad213 37051764 PMC10290870

[41] Romani GL Rossini P. Neuromagnetic functional localization: principles, state of the art, and perspectives. Brain Topogr. (1988) 1(1):5–21. 10.1007/BF01129335 3079264

[42] Hämäläinen M Hari R Ilmoniemi RJ Knuutila J Lounasmaa OV. Magnetoencephalography—theory, instrumentation, and applications to noninvasive studies of the working human brain. Rev Mod Phys. (1993) 65(2):413–97. 10.1103/RevModPhys.65.413

[43] Williamson SJ Romani GL Kaufman L Modena I, editors. Biomagnetism: an interdisciplinary approach. Series A: Life Sciences, Vol. 99. New York: Plenum Press (1982). p. vii–viii.

[44] Nenonen J Iimoniemi RJ Biomag KT. Proceedings of the 12th International Conference on Biomagnetism; August 13–17, 2000, Espoo, Finland. City ESPOO, Helsinki: Helsinki University of Technology (2001).

[45] Nowak H Haueisen J Gießler F Biomag HR. Proceedings of the 13th International Conference on Biomagnetism; August 10–14, 2002; Jena, Germany. Jena: VDE Verlag (2002). Available at: http://id.ndl.go.jp/bib/000004079562. (last accessed 02 July 2023)

[46] Halgren E Ahlfors S Haimalainen M Biomag CD. (2004). Proceedings of the 14th International Conference on Biomagnetism; August 8–12, 2004; Boston, MA. Boston: Biomag Limited (2004). Available at: https://books.google.it/books?id=7XxfjwEACAAJ.

[47] Dutz S Bellemann ME Leder U Haueisen J. Passive vortex currents in magneto- and electrocardiography: comparison of magnetic and electric signal strengths. Phys Med Biol. (2006) 51(1):145–51. 10.1088/0031-9155/51/1/011 16357437

[48] Achenbach S Moshage W. Magnetocardiography: clinical investigations with a biomagnetic multichannel system. Physiol Meas. (1993) 14(4A):A61–8. 10.1088/0967-3334/14/4A/011 8274987

[49] Makijarvi M Nenonen J Toivonen L Montonen J Katila T Siltanen P. Magnetocardiography: supraventricular arrhythmias and preexcitation syndromes. Eur Heart J. (1993) 14(suppl E):46–52. 10.1093/eurheartj/14.suppl-7B_-7De.46 8223755

[50] Lim HK Kwon H Chung N Ko Y-G Kim J-M Kim I-S Usefulness of magnetocardiogram to detect unstable angina pectoris and non-ST elevation myocardial infarction. Am J Cardiol. (2009) 103(4):448–54. 10.1016/j.amjcard.2008.10.013 19195500

[51] Bang W Kim K Lee Y Kwon H Park Y Pak H Repolarization heterogeneity of magnetocardiography predicts long-term prognosis in patients with acute myocardial infarction. Yonsei Med J. (2016) 57(6):1339–46. 10.3349/ymj.2016.57.6.1339 27593860 PMC5011264

[52] Mäkijärvi M. Magnetocardiography and cardiac risk. Herzschrittmacherther Elektrophysiol. (1997) 8(3):178–83. 10.1007/BF03042400 19484514

[53] Korhonen P Husa T Tierala I Väänänen H Mäkijärvi M Katila T Increased intra-QRS fragmentation in magnetocardiography as a predictor of arrhythmic events and mortality in patients with cardiac dysfunction after myocardial infarction. J Cardiovasc Electrophysiol. (2006) 17(4):396–401. 10.1111/j.1540-8167.2005.00332.x 16643362

[54] Comani S Liberati M Mantini D Gabriele E Brisinda D Di Luzio S Characterization of fetal arrhythmias by means of fetal magnetocardiography in three cases of difficult ultrasonographic imaging. Pacing Clin Electrophysiol. (2004) 27(12):1647–55. 10.1111/j.1540-8159.2004.00699.x 15613129

[55] Nenonen J Pesola K Hänninen H Lauerma K Takala P Mäkelä T Current-density estimation of exercise-induced ischemia in patients with multivessel coronary artery disease. J Electrocardiol. (2001) 34(Suppl):37–42. 10.1054/jelc.2001.28824 11781934

[56] Hailer B Van Leeuwen P. Clinical application of MCG in ischemic heart disease. Int congr ser. (2007):741–4. 10.1016/j.ics.2007.01.045

[57] Park JW Leithäuser B Jung F. Magnetocardiography predicts coronary artery disease in bundle-branch block patients with acute chest pain. J Electrocardiol. (2007) 40(1 Suppl):312–23. 10.1111/j.1542-474X.2005.00634.x PMC6932599 16029382

[58] Weismuller P Abraham-Fuchs K Schneider S Richter P Kochs M Edrich J Biomagnetic noninvasive localization of accessory pathways in Wolff-Parkinson-White syndrome. Pacing Clin Electrophysiol. (1991) 14(11):1961–5. 10.1111/j.1540-8159.1991.tb02798.x 1721207

[59] Brockmeier K Schmitz L De Jesus Bobadilla Chavez J Burghoff M Koch H Zimmermann RTL. Magnetocardiography and 32-lead potential mapping. J Cardiovasc Electrophysiol. (1997) 8(6):615–26. 10.1111/j.1540-8167.1997.tb01824.x 9209962

[60] Endt P Hahlbohm HD Kreiseler D Oeff M Steinhoff U Trahms L. Fragmentation of bandpass-filtered QRS-complex of patients prone to malignant arrhythmia. Med Biol Eng Comput. (1998) 36(6):723–8. 10.1007/BF02518875 10367463

[61] Oeff M Burgoff M. Magnetocardiographic localization of the origin of ventricular ectopic beats. Pacing Clin Electrophysiol. (1994) 17(3):517–22. 10.1111/j.1540-8159.1994.tb01420.x 7513881

[62] Gödde P Agrawal R Müller HP Czerki K Endt P Steinhoff U Magnetocardiographic mapping of QRS fragmentation in patients with a history of malignant tachyarrhythmias. Clin Cardiol. (2001) 24(10):682–8. 10.1002/clc.4960241009 11594414 PMC6654772

[63] Van Leeuwen P Hailer B Lange S Klein A Geue D Seybold K Quantification of cardiac magnetic field orientation during ventricular de- and repolarization. Phys Med Biol. (2008) 53(9):2291–301. 10.1088/2F0031-9155-2F53-2F9-2F006 18401064

[64] Kleemann T Kouraki K Strauss M Skarlos A Zeymer U Zahn R. Prognostic value of electromagnetic QRS fragmentation in survivors of sustained ventricular tachycardia or ventricular fibrillation compared with healthy controls. J Interv Card Electrophysiol. (2013) 36(3):255–60. 10.1007/s10840-012-9754-6 23179924

[65] Lim HK Chung N Kim K Ko YG Kwon H Lee YH Reproducibility of quantitative estimate of magnetocardiographic ventricular depolarization and repolarization parameters in healthy subjects and patients with coronary artery disease. Ann Biomed Eng. (2007) 35(1):59–68. 10.1007/s10439-006-9210-9 17089073

[66] Fenici R Brisinda D Nenonen J Morana G Fenici P. First MCG multichannel instrumentation operating in an unshielded hospital laboratory for multi-modal cardiac electrophysiology: preliminary experience. Biomed Tech Eng. (2001) 46(s2):219–22. 10.1515/bmte.2001.46.s2.219

[67] Fenici R Brisinda D. First 36-channel system for clinical magnetocardiography in unshielded hospital laboratory for cardiac electrophysiology. Int J Bioelectromagn. (2003) 5(1):80–3.

[68] Fenici R Brisinda D Meloni AM Sternickel K Fenici P. Clinical validation of machine learning for automatic analysis of multichannel magnetocardiography. Lect Notes Comput Sci. (2005) 1:143–52. 10.1007/11494621_15

[69] Hailer B Van Leeuwen P Lange S Wehr M. Spatial distribution of QT dispersion measured by magnetocardiography under stress in coronary artery disease. J Electrocardiol. (1999) 32(3):207–16. 10.1016/S0022-0736(99)90103-6 10465564

[70] Brisinda D Meloni AM Nenonen J Giorgi A Fenici R. First 36-channel magnetocardiographic study of patients with coronary artery disease in an unshielded laboratory. Biomed Tech. (2004) BAND 48:134–6. (Ergänzungsband 2).

[71] Chaikovsky I Hailer B Sosnytskyy V Lutay M Mjasnikov G Kazmirchuk A Predictive value of the complex magnetocardiographic index in patients with intermediate pretest probability of chronic coronary artery disease: results of a two-center study. Coron Artery Dis. (2014) 25(6):474–84. 10.1097/MCA.0000000000000107 24667125

[72] Tolstrup K Madsen BE Ruiz JA Greenwood SD Camacho J Siegel RJ Non-invasive resting magnetocardiographic imaging for the rapid detection of ischemia in subjects presenting with chest pain. Cardiology. (2006) 106(4):270–6. 10.1159/000093490 16733351

[73] Steinberg BA Roguin A Watkins SP Hill P Fernando D Resar JR. Magnetocardiogram recordings in a nonshielded environment—reproducibility and ischemia detection. Ann Noninvasive Electrocardiol. (2005) 10(2):152–60. 10.1111/j.1542-474X.2005.05611.x 15842427 PMC6932464

[74] Kashyap R Smars PA Gartzen C Bellolio MFRG. Incremental value of magnetocardiography over electrocardiography in diagnosing acute coronary syndrome in patients presenting to the emergency department with high risk unstable angina. Ann Emerg Med. (2008) 51:521. 10.1016/j.annemergmed.2008.01.133

[75] Escalona-Vargas D Bolin EH Lowery CL Siegel ER Eswaran H. Recording and quantifying fetal magnetocardiography signals using a flexible array of optically-pumped magnetometers. Physiol Meas. (2020) 41(12):1–14. 10.1088/1361-6579/abc353 PMC7875519 33086201

[76] Gapelyuk A Wessel N Fischer R Zacharzowsky U Koch L Selbig D Detection of patients with coronary artery disease using cardiac magnetic field mapping at rest. J Electrocardiol. (2007) 40(5):401–7. 10.1016/j.jelectrocard.2007.03.013 17531250

[77] Schirdewan A Gapelyuk A Fischer R Koch L Schütt H Zacharzowsky U Cardiac magnetic field map topology quantified by Kullback-Leibler entropy identifies patients with hypertrophic cardiomyopathy. Chaos. (2007) 17(1):1–10. 10.1063/1.2432059 17411275

[78] Chaikovsky I Primin M Nedayvoda IBM. Magnetocardiography in unshielded setting: heart electrical image based on 2-D and 3-D data in comparison with perfusion image based on PET results clinical cases. In: Chaikovsky I, editor. Coronary artery diseases. Kyiv, Ukraine: InTech (2012). p. 43–58.

[79] Fenici RR Melillo G. Biomagnetically localizable multipurpose catheter and method for MCG guided intracardiac electrophysiology, biopsy and ablation of cardiac arrhythmias. Int J Card Imaging. (1991) 7(3–4):207–2015. 10.1007/BF01797753 1820402

[80] Fenici RR Melillo G Masselli M. Clinical magnetocardiography—10 years experience at the catholic university. Int J Card Imaging. (1991) 7(3–4):151–67. 10.1007/BF01797748 1820397

[81] Wu YW Lee CM Liu YB Wang SS Huang HC Tseng WK Usefulness of magnetocardiography to detect coronary artery disease and cardiac allograft vasculopathy. Circ J. (2013) 77(7):1783–90. 10.1253/circj.CJ-12-1170 23603823

[82] Fenici R Brisinda D Venuti A Sorbo AR. Thirty years of clinical magnetocardiography at the Catholic University of Rome: diagnostic value and new perspectives for the treatment of cardiac arrhythmias. Int J Cardiol. (2013) 168(5):5113–5. 10.1016/j.ijcard.2013.07.238 23958422

[83] Gussmann AW Strasburger JF Wakai RT Strasburger JF. Contribution of fetal magnetocardiography to diagnosis, risk assessment, and treatment of fetal arrhythmia. J Am Heart Assoc. (2022) 11(15):1–11, e025224. 10.1161/JAHA.121.025224 PMC9375504 35904205

[84] Brisinda D Caristo ME Fenici R. Magnetocardiographic estimate of cardiac intervals in guinea pigs. Comparison between conscious and anesthetized conditions. Int Congr Ser. (2007) 1300:447–50. 10.1016/j.ics.2006.12.047

[85] Jensen K Skarsfeldt MA Stærkind H Arnbak J Balabas MV Olesen SP Magnetocardiography on an isolated animal heart with a room-temperature optically pumped magnetometer. Sci Rep. (2018) 8(1):1–9. 10.1038/s41598-018-34535-z 30385784 PMC6212485

[86] McBride KK Roth BJ Sidorov VY Wikswo JP Baudenbacher FJ. Measurements of transmembrane potential and magnetic field at the apex of the heart. Biophys J. (2010) 99(10):3113–8. 10.1016/j.bpj.2010.08.040 21081057 PMC2980705

[87] Brisinda D Fenici R Smars P. New technologies for the evaluation of acute coronary syndromes: magnetocardiography—the next generation of super electrocardiogram? In: Pena M Osborne A Peacock WF, editors. Short stay management of_chest pain. 2nd ed. Humana Press (Contemporary Cardiology) (2022). p. 177–2013. 10.1007/978-3-031-05520-1_17

[88] Camm AJ Henderson R Brisinda D Body R Charles RG Varcoe B Clinical utility of magnetocardiography in cardiology for the detection of myocardial ischemia. J Electrocardiol. (2019) 57:10–7. 10.1016/j.jelectrocard.2019.07.009 31442563

[89] Jensen K Bentzen BH Polzik ES. Small animal biomagnetism applications. In: Labyt E Sander T Wakai R, editors. Flexible high performance magnetic field sensors. Cham: Springer International Publishing (2022). p. 33–48. Available at: https://link.springer.com/10.1007/978-3-031-05363-4_3. (last accessed 02 July 2023)

[90] Kwong JSW Leithäuser B Park J-W Yu C-M. Diagnostic value of magnetocardiography in coronary artery disease and cardiac arrhythmias: a review of clinical data. Int J Cardiol. (2013) 167(5):1835–42. 10.1016/j.ijcard.2012.12.056 23336954

[91] Rassi D Zhuravlev YE Lewis MJ Emery SJ. Vector fetal magnetocardiography. In: Aine CJ Stroink G Wood CC Okada Y Swithenby SJ, editors. Biomag 96. New York, NY: Springer New York (2000). p. 533–6.

[92] Carré F Painvin I Poiseau E Mabo P Lessard Y Daubert JC Magnetocardiography: principles and potential uses in cardiology. Arch Mal Coeur Vaiss. (2002) 95(10):924–32. 12462903

[93] Stroink G Moshage W Achenbach S. Magnetism in medicine: a handbook, chapter cardiomagnetism. New York: Wiley-VCH (1998).

[94] Stroink G. Forty years of magnetocardiology BT. In: Supek S Sušac A, editors. 17th international conference on biomagnetism advances in biomagnetism—biomag2010. Berlin: Springer (2010). p. 1–8.

[95] Sternickel K Braginski AI. Biomagnetism using SQUIDs: status and perspectives. Supercond Sci Technol. (2006) 19(3):S160–S71. 10.1088/0953-2048/19/3/024

[96] Udovychenko Y Popov A Chaikovsky I. Multistage classification of current density distribution maps of various heart states based on correlation analysis and k-NN algorithm. Front Med Technol. (2021) 3(December):1–9. 10.3389/fmedt.2021.779800 PMC8757770 35047968

[97] Anogianakis G Badier JM Barrett G Erné S Fenici R Fenwick P A consensus statement on relative merits of EEG and MEG. Electroencephalogr Clin Neurophysiol. (1992) 82(5):317–9. 10.1016/0013-4694(92)90001-X 1374700

[98] Fenici RR. Clinical assessment of the magnetocardiogram. In: Williamson SJ Romani GL Kaufman L MI, editors. Biomagnetism: an interdisciplinary approach. NATO ASI S. New York: Plenum Press (1982). p. 285–98.

[99] Cheyne D. New frontiers in biomagnetism. *Proceedings of the 15th International Conference on Biomagnetism*; August 21–25, 2006; Vancouver, BC, Canada. Vancouver: Elsevier (2007). (International congress series). Available at: https://books.google.it/books?id=s8pqAAAAMAAJ. (last accessed 02 July 2023)

[100] Siltanen P. Magnetocardiography. Vol. 2. New York: Pergamon Press (1989). p. 1405–38.

[101] Tavarozzi I Comani S Del Gratta C Di Luzio S Romani GL Gallina S Magnetocardiography: current status and perspectives. Part II: clinical applications. Ital Heart J. (2002) 3(3):151–65. 11974660

[102] Fenici R Brisinda D Meloni AM. Clinical application of magnetocardiography. Expert Rev Mol Diagn. (2005) 5(3):291–313. 10.1586/14737159.5.3.291 15934809

[103] Yamada S Yamaguchi I. Magnetocardiograms in clinical medicine: unique information on cardiac ischemia, arrhythmias, and fetal diagnosis. Intern Med. (2005) 44(1):1–19. 10.2169/internalmedicine.44.1 15704657

[104] Rogalla H Kes PH. 100 years of superconductivity. Boca Raton, FL: CRC Press (2012). p. 2012.

[105] Mäkijärvi M Korhonen P Jurkko R Väänänen H Siltanen P Hänninen H. Magnetocardiography. In: Macfarlane PW van Oosterom A Pahlm O Kligfield P Janse M Camm J, editors. Comprehensive electrocardiology. London: Springer London (2010). p. 2007–28. Available at: 10.1007/978-1-84882-046-3_44. (last accessed 02 July 2023)

[106] Cohen D Norman JC Molokhia F Hood W. Magnetocardiography of direct currents: S-T segment and baseline shifts during experimental myocardial infarction. Science. (1971) 172(3990):1329–33. 10.1126/science.172.3990.1329 5580214

[107] Savard P Cohen D Lepeschkin E Cuffin BN Madias JE. Magnetic measurement of S-T and T-Q segment shifts in humans. Part I: early repolarization and left bundle branch block. Circ Res. (1983) 53(2):264–73. 10.1161/01.RES.53.2.264 6883649

[108] Cohen D Savard P Rifkin RD. Magnetic measurements of S-T and T-Q segment shifts in humans. Part II: exercise-induced S-T segment depression. Circ Res. (1983) 53(2):274–9. 10.1161/01.RES.53.2.274 6883650

[109] Kariniemi V Ahopelto J Karp PJ Katila TE. The fetal magnetocardiogram. J Perinat Med. (1974) 2(3):214–6. 10.1515/jpme.1974.2.3.214 4468305

[110] Karp PJ Katila TE Saarinen M Siltanen P Varpula TT. The normal human magnetocardiogram. II. A multipole analysis. Circ Res. (1980) 47(1):117–30. 10.1161/01.RES.47.1.117 7379262

[111] Seki Y Kandori A Kumagai Y Ohnuma M Ishiyama A Ishii T Unshielded fetal magnetocardiography system using two-dimensional gradiometers. Rev Sci Instrum. (2008) 79(3):36103–6. 10.1063/1.2897588 18377051

[112] Fujino K Sumi M Saito K Murakami M Higuchi T Nakaya Y Magnetocardiograms of patients with left ventricular overloading recorded with a second-derivative SQUID gradiometer. J Electrocardiol. (1984) 17(3):219–28. 10.1016/S0022-0736(84)80058-8 6237164

[113] Takeuchi A Watanabe K Nomura M Ishihara S Sumi M Murakami M The P wave in the magnetocardiogram. J Electrocardiol. (1988) 21(2):161–7. 10.1016/S0022-0736(88)80012-8 3397699

[114] Kotani M Mori H Kuriki S Uchikawa Y Chiyotani K Nemoto I. Biomagnetism in Japan. Med Prog Technol. (1987) 12(3–4):233–42. 10.1007/978-94-009-3361-3_20 3627030

[115] Fenici RR Romani GL Leoni R. Magnetocardiographic recording of the His-Purkinje system activity in man. Jpn Heart J. (1982) 23(Suppl. 1):728–30.

[116] Farrell DE Tripp JHNR. Non-invasive information of the PR segment of the cardiac cycle. An assessment of the clinical potential of the electric and magnetic methods. Proc SPIE. (1978) 167:173.

[117] Farrell DE Tripp JH VanDoren CL. High resolution cardiomagnetism. In: Erné SN Hahlbohm H-D Lübbig H, editors. Proceedings of the Third International Workshop; Berlin (West); May 1980. Boston, MA: De Gruyter (1981). p. 273–82. Available at: 10.1515/9783110863529-019. (last accessed 02 July 2023)

[118] Farrell DE Tripp JH Norgren R. Magnetic study of the His-Purkinje conduction system in man. IEEE Trans Biomed Eng. (1980) BME-27(7):345–50. 10.1109/TBME.1980.326646 7409799

[119] Tripp JH Farrell DE. Theory of the Pr sequent of the human magnetocardiogram. In: Erné SN Hahlbohm H-D Lübbig H, editors. Proceedings of the third international workshop; Berlin (West); May 1980. Berlin, New York: De Gruyter (1981). p. 259–72. Available at: 10.1515/9783110863529-018. (last accessed 02 July 2023)

[120] Fenici RR Romani GL Erné SN. High-resolution magnetic measurements of human cardiac electrophysiological events. Nuovo Cim D. (1983) 2(2):231–47. 10.1007/BF02455927

[121] Leifer M Capos N Griffin J Wikswo J. Atrial activity during the PR segment of the MCG. Nuovo Cim D. (1983) 2(2):266–79. 10.1007/BF02455930

[122] Patrick JL Hess DW Tripp JH Farrell DE. The magnetic field produced by the conduction system of the human heart. Nuovo Cim D. (1983) 2(2):255–65. 10.1007/BF02455929

[123] Fenici RR Masselli M Ernè SNHH. Magnetocardiographic mapping of the P-R interval phenomena in an unshielded hospital laboratory. In: Weinberg H Stroink G, editors. Biomagnetism, application and theory. New York: Pergamon Press (1985). p. 137–41.

[124] Erne SN Lehmann HP Masselli M Uchikawa Y. Modelling of the His-Purkinje heart conduction system. In: Weinberg H Stroink G, editors. Biomagnetism, application and theory. New York: Pergamon Press (1985). p. 126.

[125] Uchikawa Y Kotani M Erne S. Study of P-R segment using magnetocardiographic measurement. Magn Japan IEEE Transl J. (1987) 2:851–2. 10.1109/TJMJ.1987.4549630

[126] Fenici RR Melillo G. Biomagnetic imaging in the cardiac catheterization laboratory. In: Williamson SJ Hoke M Stroink G Kotani M, editors. Advances in biomagnetism. Boston, MA: Springer US (1989). p. 409–15. Available at: 10.1007/978-1-4613-0581-1_87. (last accessed 02 July 2023)

[127] Fenici RR Masselli M Lopez L Sabetta F. First simultaneous magnetocardiographic and invasive recordings of the PR interval electrophysiological phenomena in man. Med Biol Eng Comput. (1985) 23(Suppl):1483–4.

[128] Fenici R Brisinda D. Bridging noninvasive and interventional electroanatomical imaging: role of magnetocardiography. J Electrocardiol. (2007) 40(1 Suppl):47–52. 10.1016/j.jelectrocard.2006.10.03217027018

[129] Yamada S Kuga K On K Yamaguchi I. Noninvasive recording of his potential using magnetocardiograms. Circ J. (2003) 67(7):622–4. 10.1253/circj.67.622 12845187

[130] Senthilnathan S Chandrasekaran P Narayanan M Patel R Katholil G Janawadkar MP Enhancing the reliability in the noninvasive measurement of the his bundle magnetic field using a novel signal averaging methodology. Ann Noninvasive Electrocardiol. (2012) 17(3):186–94. 10.1111/j.1542-474X.2012.00523.x 22816537 PMC6932326

[131] Sengottuvel S Raja JS Rajesh P Santhosh S Gireesan K Madhukar PJ Noninvasive determination of HV interval using magnetocardiography. Pacing Clin Electrophysiol. (2017) 40:568–77. 10.1111/pace.13067 28247926

[132] Nenonen JT. Solving the inverse problem in magnetocardiography. IEEE Eng Med Biol Mag. (1994) 13(4):487–96. 10.1109/51.310989

[133] Nenonen J Purcell CJ Horacek BM Stroink G Katila T. Magnetocardiographic functional localization using a current dipole in a realistic torso. IEEE Trans Biomed Eng. (1991) 38(7):658–64. 10.1109/10.83565 1879858

[134] Dössel O. Inverse problem of electro- and magnetocardiography: review and recent progress. Int J Bioelectromagn. (2000) 2(2):262–85. Available at: http://ijbem.k.hosei.ac.jp/volume2/number2/doessel/paper_ijbem.htm-5Cnhttp://www.ijbem.org/volume2/number2/doessel/paper_ijbem.htm. (last accessed 02 July 2023)

[135] Rudy Y Messinger-Rapport BJ. The inverse problem in electrocardiography: solutions in terms of epicardial potentials. Crit Rev Biomed Eng. (1988) 16(3):215–68. 3064971

[136] Gulrajani RM Savard P Roberge FA. The inverse problem in electrocardiography: solutions in terms of equivalent sources. Crit Rev Biomed Eng. (1988) 16(3):171–214. Available at: http://www.ncbi.nlm.nih.gov/pubmed/3064970. (last accessed 02 July 2023) 3064970

[137] Fenici R Covino M Masselli M Melillo G. Magnetocardiographic mapping of reentry tachycardias in high resolution electrocardiography. In: El-Sherif N Turitto G, editors. High resolution electrocardiography 1994 update. Bologna: Moduzzi Editore (1994). p. 73–8.

[138] Fenici R Melillo G. Magnetocardiography: ventricular arrhythmias. Eur Heart J. (1993) 14(Suppl E):53–60. 10.1093/eurheartj/14.suppl_E.53 8223756

[139] Fenici RR Melillo G. Biomagnetic imaging in the cardiac catheterization laboratory. In: Williamson SJ Hoke M Stroink G Kotani M, editors. Advances in biomagnetism. Boston, MA: Springer US (1989). p. 409–15.

[140] Erné SN Fenici RR Hahlbohm H-D Jaszczuk W Lehmann HP Masselli M. High-resolution magnetocardiographic recordings of the ST segment in patients with electrical late potentials. Nuovo Cim D. (1983) 2(2):340–5. 10.1007/BF02455936

[141] Fenici RR Melillo G. Biomagnetic study of cardiac arrhythmias. Clin Phys Physiol Meas. (1991) 12:5–10. 10.1088/0143-0815/12/A/001 1778053

[142] Fenici RR Masselli M Lopez LMM. High-resolution electrocardiography and magnetocardiography. In: El-Sherif NTG, editor. High-resolution electrocardiography. Mount Kisko, NY: Futura Publishing (1992). p. 147–71.

[143] Fenici R Melillo G Cappelli A De Luca C Masselli M. Atrial and ventricular tachycardias: invasive validation and reproducibility of magnetocardiographic imaging. In: Williamson SJ Hoke M Stroink G Kotani M, editors. Advances in biomagnestism. New York: Plenum Press (1989). p. 441–4. Available at: 10.1007/978-1-4613-0581-1_94. (last accessed 02 July 2023)

[144] Fenici R Masselli M Sabetta F. Simultaneous magnetocardiographic mapping and invasive electrophysiology to evaluate the accuracy of the equivalent current dipole inverse solution for the localization of human cardiac sources. New Trends Arrhythm. (1986) 2(2):357–71.

[145] Fenici RR Melillo G Cappelli A De Luca C Masselli M. Magnetocardiographic localization of a pacing catheter. In: Williamson SJ Hoke M Stroink G Kotani M, editors. Advances in biomagnetism. New York: Springer (1989). p. 361–4.

[146] Fenici R. Elettrocatetere multifunzioni, localizzabile magneticamente per registrazione di potenziali monofasici cardiaci ed ablazione endocavitaria di strutture aritmogene Brevetto no. 1219855. Italy: Ministero dell’Industra del Commercio e dell’Artigianato. 1219855(1989).

[147] Fenici R. Biomagnetically localizable multipurpose catheter and method for magnetocardiographic guided intracardiac mapping, biopsy, and ablation of cardiac arrhythmias. Italy: United States Patent. US 5056517(1991). 10.1007/BF01797753 1820402

[148] Fenici R. Intracardiac catheter, magnetocardiographically localizable, for mapping and pacing provided with means for ablation of arrhythmogenic tissue. Italy: European Patent Office. 0428 812 A1(1991).

[149] Fenici RR Covino M Cellerino C Di Lillo M De Filippo MC Melillo G. Magnetocardiographically-guided catheter ablation. J Interv Cardiol. (1995) 8(6 Suppl):825–36. 10.1111/j.1540-8183.1995.tb00936.x 10159774

[150] Fenici RR. Biomagnetic imaging for ablation of cardiac arrhythmias (editorial). Int J Cardiac Imaging (1991) 7(3–4):iii.

[151] Schneider S Hoenig E Reichenberger H Abraham-Fuchs K Moshage W Oppelt A Multichannel biomagnetic system for study of electrical activity in the brain and heart. Radiology. (1990) 176(3):825–30. 10.1148/radiology.176.3.2389043 2389043

[152] Moshage W Achenbach S Weikl A Göhl K Bachmann K Abraham-Fuchs K Clinical magnetocardiography: experience with a biomagnetic multichannel system. Int J Card Imaging. (1991) 7(3–4):217–23. 10.1007/BF01797754 1726471

[153] Moshage W Achenbach S Göhl K Bachmann K. Evaluation of the non-invasive localization accuracy of cardiac arrhythmias attainable by multichannel magnetocardiography. Int J Card Imaging. (1996) 12(1):47–59. 10.1007/bf01798116 8847454

[154] Sheet F. Birch-large-scale facility in biomagnetism (1998). p. 1994–5. Available at: https://cordis.europa.eu/project/id/CHGE940068. (last accessed 02 July 2023)

[155] Fenici R Pesola K Mäkijärvi M Nenonen J Teener U Fenici P Nonfluoroscopic localization of an amagnetic catheter in a realistic torso phantom by magnetocardiographic and body surface potential mapping. Pacing Clin Electrophysiol. (1998) 21(11 Pt 2):2485–91. 10.1111/j.1540-8159.1998.tb01206.x 9825372

[156] Fenici R Nenonen J Pesola K Korhonen P Lötjönen J Mäkijärvi M Nonfluoroscopic localization of an amagnetic stimulation catheter by multichannel magnetocardiography. Pacing Clin Electrophysiol. (1999) 22(8):1210–20. 10.1111/j.1540-8159.1999.tb00602.x 10461298

[157] Fenici R Pesola K Korhonen P Mäkijärvi M Nenonen J Toivonen L Magnetocardiographic pacemapping for nonfluoroscopic localization of intracardiac electrophysiology catheters. Pacing Clin Electrophysiol. (1998) 21(11 Pt 2):2492–9. 10.1111/j.1540-8159.1998.tb01207.x 9825373

[158] Fenici R. Improved amagnetic catheter and method for single-catheter multiple monophasic action potential recording. Tridimensionally localizable and guided onto the arrhytmogenic substrate by body surface magnetocardiographic mapping. Italy: Australian Patent Office. AUS 2000116803 B2(2000).

[159] Fenici R. Catheter guidance by magnetocardiographic mapping. Vol. 1. Italy. United States Patent. US 6,527,724 B1(2003).

[160] Pesola K Nenonen J Fenici RKT. Comparison of regularization methods when applied to epicardial minimum norm estimates. Biomed Tech. (1997) 42(S1):273–6.

[161] Pesola K Nenonen J Fenici R Lötjönen J Mäkijärvi M Fenici P Bioelectromagnetic localization of a pacing catheter in the heart. Phys Med Biol. (1999) 44(10):2565–78. 10.1088/0031-9155/44/10/314 10533929

[162] Pesola K Lötjönen J Nenonen J Magnin IE Lauerma K Fenici R The effect of geometric and topologic differences in boundary element models on magnetocardiographic localization accuracy. IEEE Trans Biomed Eng. (2000) 47(9):1237–47. 10.1109/10.867958 11008425

[163] Pesola K Nenonen J. Current density imaging on the epicardial surface of the heart. Geology. (2000):0–3. Available from: https://www.semanticscholar.org/paper/Current-density-imaging-on-the-epicardial-surface-Pesola-Nenonen/4ce42520bc24ef501b76c6076d58054c9e7d82f9

[164] Leder U Haueisen J Huck M Nowak H. Non-invasive imaging of arrhythmogenic left-ventricular myocardium after infarction. Lancet. (1998) 352(9143):1825. 10.1016/S0140-6736(98)00082-8 9851385

[165] Nenonen J Pesola K Fenici R Lauerma K Makijarvi M Katila T. Current density imaging of focal cardiac sources. Biomed Tech Eng. (2001) 46(s2):50–3. 10.1515/bmte.2001.46.s2.50

[166] Fenici R Brisinda D Pesola K Nenonen J Fenici P Katila T. Validation of magnetocardiographic current density imaging with a non-magnetic stimulation catheter. In: Proceedings of the 12th international conference on biomagnetism. Helsinki: BIOMAG CENTRAL -ark:/13960/t5q87v12v (2001). p. 839–42. Available at: http://citeseerx.ist.psu.edu/viewdoc/download?doi=10.1.1.5.7244&rep=rep1&type=pdf. (last accessed 02 July 2023)

[167] Gepstein L Evans SJ. Electroanatomical mapping of the heart: basic concepts and implications for the treatment of cardiac arrhythmias. Pacing Clin Electrophysiol. (1998) 21(6):1268–78. 10.1111/j.1540-8159.1998.tb00187.x 9633070

[168] Gepstein L Hayam G Ben-Haim SA. A novel method for nonfluoroscopic catheter-based electroanatomical mapping of the heart in vitro and in vivo accuracy results. Circulation. (1997) 95(6):1611–22. 10.1161/01.CIR.95.6.1611 9118532

[169] Kirchhof P Loh P Eckardt L Ribbing M Rolf S Eick O A novel nonfluoroscopic catheter visualization system (LocaLisa) to reduce radiation exposure during catheter ablation of supraventricular tachycardias. Am J Cardiol. (2002) 90(3):340–3. 10.1016/S0002-9149(02)02481-5 12127630

[170] Wittkampf FHM Wever EFD Derksen R Wilde AAM Ramanna H Hauer RNW Localisa: new technique for real-time 3-dimensional localization of regular intracardiac electrodes. Circulation. (1999) 99(10):1312–7. 10.1161/01.cir.99.10.1312 10077514

[171] Taccardi B. Body surface mapping and cardiac electric sources: a historical survey. J Electrocardiol. (1990) 23:150–4. 10.1016/0022-0736(90)90091-F 2090733

[172] Medvegy M Duray G Pintér A Préda I. Body surface potential mapping: historical background, present possibilities, diagnostic challenges. Ann Noninvasive Electrocardiol. (2002) 7(2):139–51. 10.1111/j.1542-474X.2002.tb00155.x 12049686 PMC7027621

[173] Fenici RR Brisinda D Fenici P Morana G Ruggieri MP. Multimodal cardiac imaging in the clinical electrophysiology laboratory. Proceedings of the 12th international conference on biomagnetism. Helsinki: BIOMAG CENTRAL -ark:/13960/t5q87v12v (2001). p. 542–5.

[174] Bourier F Reents T Ammar-Busch S Buiatti A Grebmer C Telishevska M Sensor-based electromagnetic navigation Mediguide®: how accurate is it? A phantom model study. J Cardiovasc Electrophysiol. (2015) 26(10):1140–5. 10.1111/jce.12741 26086594

[175] Fenici R Brisinda D Nenonen J Morana G P. Fenici. First MCG multichannel instrumentation operative in an unshielded hospital laboratory for multimodal cardiac electrophysiology. Preliminary experience. Biomed Tech. (2001) Band 46:2019–222.

[176] Fenici R Brisinda D Nenonen J. Multimodal integration of MAP recordings and MCG imaging in patients with paroxysmal atrial arrhythmias, using the MultiMAP amagnetic catheter. In: Nowak H Haueisen J Gießler F Huonker R, editors. Proceedings of the 13th international conference on biomagnetism. Berlin: VDE Verlag GMBH; (2002). p. 518–20.

[177] Fenici R Sorbo AR Brisinda D. Contactless three-dimensional electro-anatomical imaging based on magnetocardiography is reliable in unshielded hospital environments: a retrospective study of 635 patients. Eur Heart J. (2018) 39(suppl_1):ehy565.P2269. 10.1093/eurheartj/ehy565.P2269

[178] Veisterä H Fenici R Lötjönen J. Online heart model creation for magnetocardiographic measurements. Proceedings of 14th international conference on biomagnetism. Boston, Massachusetts, USA: BioMag 2004 Ltd (2004). p. 415–6.

[179] Nakai K Izumoto H Kawazoe K Tsuboi J Fukuhiro Y Oka T Three-dimensional recovery time dispersion map by 64-channel magnetocardiography may demonstrate the location of a myocardial injury and heterogeneity of repolarization. Int J Cardiovasc Imaging. (2006) 22(3–4):573–80. 10.1007/s10554-005-9019-x 16307313

[180] Nakai K Kazui T Okabayashi H Hayashi R Fukushima A Suwabe A. Development of three-dimensional analysis of current density distribution by 64-ch magnetocardiography and clinical application. Rinsho Byori. (2008) 56(12):1118–24. 19175077

[181] Nakai K Takanori O Hitoshi O Junichi T Yoshiaki F Akimune F Three-dimensional spectral map of atrial fibrillation by a 64-channel magnetocardiogram. J Electrocardiol. (2008) 41(2):123–30. 10.1016/j.jelectrocard.2007.06.008 17884079

[182] Kim D Kim K Lee Y-H Ahn H. Detection of atrial arrhythmia in superconducting quantum interference device magnetocardiography preliminary result of a totally-noninvasive localization method for atrial current mapping. Interact Cardiovasc Thorac Surg Thorac Surg. (2007) 6(3):274–9. 10.1510/icvts.2006.142869 17669841

[183] Fenici R Brisinda D. Magnetocardiography provides non-invasive three-dimensional electroanatomical imaging of cardiac electrophysiology. Anadolu Kardiyol Derg. (2007) 7(Suppl 1):23–8. 10.1007/s10554-006-9076-9 17584673

[184] Brisinda D Meloni AM Fenici R. First 36-channel magnetocardiographic study of CAD patients in an unshielded laboratory for interventional and intensive cardiac care. Funct Imaging Model Hear. (2003) LNCS 2674:122–31. 10.1007/3-540-44883-7_13

[185] Brisinda D Comani S Meloni AM Alleva G Mantini D Fenici R. Multichannel mapping of fetal magnetocardiogram in an unshielded hospital setting. Prenat Diagn. (2005) 25(5):376–82. 10.1002/pd.1160 15906428

[186] Brisinda D Meloni AM Fenici R. Clinical multichannel MCG in unshielded hospital environment. Neurol Clin Neurophysiol. (2004) 8:1–8. 16015715

[187] Brisinda D Meloni A Fenici P Fenici R. Unshielded multichannel magnetocardiographic study of ventricular repolarization abnormalities in patients with mitral valve prolapse. Biomed Tech. (2004) 48(2):128–30.

[188] Fenici R Brisinda D Meloni AM. Effects of filtering on computer-aided analysis for detection of chronic ischemic heart disease with unshielded rest magnetocardiography mapping. Neurol Clin Neurophysiol. (2004) 7:1–5. 16012666

[189] Sorbo AR Lombardi G La Brocca L Guida G Fenici R Brisinda D. Unshielded magnetocardiography: repeatability and reproducibility of automatically estimated ventricular repolarization parameters in 204 healthy subjects. Ann Noninvasive Electrocardiol. (2018) 23(3):1–12. 10.1111/anec.12526 PMC6931803 29266621

[190] Brisinda D Fenici R. Magnetocardiographic study of patients with right and left bundle branch blocks. Int Congr Ser. (2007) 1300:451–4. 10.1016/j.ics.2006.12.061

[191] Brisinda D Meloni AM Nenonen J Fenici R. Unshielded stress multichannel magnetocardiography of patients with coronary disease and normal subjects with standard ergometer. Comparison with ECG. Biomed Tech. (2004) Band 48(2):137–9.

[192] Fenici R Brisinda D Nenonen J Fenici P. Noninvasive study of ventricular preexcitation using multichannel magnetocardiography. Pacing Clin Electrophysiol. (2003) 26(1 Pt 2):431–5. 10.1046/j.1460-9592.2003.00064.x 12687860

[193] Brisinda D Fenici R. Noninvasive classification of ventricular preexcitation with unshielded magnetocardiography and transesophageal atrial pacing and follow-up. Pacing Clin Electrophysiol. (2007) 30(Suppl. 1):S1–5. 10.1111/j.1540-8159.2007.00627.x 17302694

[194] Fenici RR Brisinda D Mäkijärvi M Toivonen L Pesola K Nenonen JT High resolution MSI-guided multiple monophasic action potential mapping with a single amagnetic catheter. In: Nenonen J Iimoniemi RJ Katila T, editors. Biomag 2000. Helsinki: BIOMAG CENTRAL -ark:/13960/t5q87v12v (2000). p. 3–6.

[195] Brisinda D Meloni AM Fenici R. Contactless magnetocardiographic study of ventricular repolarization in intact Wistar rats: evidence of gender-related differences. Basic Res Cardiol. (2004) 99(3):193–203. 10.1007/s00395-003-0453-4 15088104

[196] Brisinda D Caristo ME Fenici R. Contactless magnetocardiographic mapping in anesthetized Wistar rats: evidence of age-related changes of cardiac electrical activity. Am J Physiol Hear CircPhysiol. (2006) 291(1):H368–78. 10.1152/ajpheart.01048.2005 16373584

[197] Brisinda D Caristo ME Fenici R. Contactless magnetocardiographic study of age- and gender-related variability of ventricular repolarization parameters in Guinea pigs. Int Congr Ser. (2007) 1300:443–6. 10.1016/j.ics.2006.12.060

[198] Brisinda D Giordano V Calvani M Fenici R. Study of ventricular repolarization changes after carnitine deprivation in rats by contactless magnetocardiography. Int Congr Ser. (2007) 1300:455–8. 10.1016/j.ics.2006.12.039

[199] Brisinda D Caristo ME Fenici R. Longitudinal study of cardiac electrical activity in anesthetized guinea pigs by contactless magnetocardiography. Physiol Meas. (2007) 28(8):773–92. 10.1088/0967-3334/28/8/002 17664671

[200] Brisinda D Sorbo AR Venuti A Fenici R. Percutaneous method for single-catheter multiple monophasic action potential recordings during magnetocardiographic mapping in spontaneously breathing rodents. Physiol Meas. (2012) 33(3):521–34. 10.1088/0967-3334/33/3/521 22373565

[201] Steinhoff U Knappe-Grueneberg S Schnabel A Trahms L Smith F Langley P Magnetocardiography for pharmacology safety studies requiring high patient throughput and reliability. J Electrocardiol. (2004) 37(Suppl):187–92. 10.1016/j.jelectrocard.2004.08.055 15534839

[202] Fischer R Gapelyuk A Wessel N Gruner K Gruner A Müller D Time course of changes in cardiac magnetic field mapping after myocardial infarction in rats. Int Congr Ser. (2007) 1300:484–7. 10.1016/j.ics.2006.12.018

[203] Fischer R Dechend R Gapelyuk A Shagdarsuren E Gruner K Gruner A Angiotensin II-induced sudden arrhythmic death and electrical remodeling. Am J Physiol Hear Circ Physiol. (2007) 293(2):1242–53. 10.1152/ajpheart.01400.2006 17416596

[204] Uchida S Iramina K Goto K Ueno S. Current source imaging for high spatial resolution magnetocardiography in normal and abnormal rat cardiac muscles. J Appl Phys. (2000) 87(9):6205. 10.1063/1.372655

[205] Komamura K. ASSA13-03-14 drug-induced QT prolongation in guinea pig is automatically analysed by micro-magnetocardiography system with superconducting quantum interference device: comparison with ECG. Heart. (2013) 99(Suppl 1):A18. Available at: https://heart.bmj.com/content/99/Suppl_1/A18.2. 10.1136/heartjnl-2013-303992.054. (last accessed 02 July 2023)

[206] Komamura K Kawai J Miyamoto M Adachi Y Uehara G Haruta Y. Diagnosis of the location of myocardial injury using mouse/rat magnetocardiography system with a single-chip SQUID magnetometer array. Int Congr Ser. (2007) 1300:574–7. 10.1016/j.ics.2006.12.062 23853129

[207] Lindseth B Schwindt P Kitching J Fischer D Shusterman V. Non-contact measurement of cardiac electromagnetic field in mice by use of a microfabricated atomic magnetometer. Comput Cardiol. (2007) 34:443–6.

[208] Arai K Kuwahata A Nishitani D Fujisaki I Matsuki R Nishio Y Millimetre-scale magnetocardiography of living rats with thoracotomy. Commun Phys. (2022) 5(1):1–10. 10.1038/s42005-022-00978-0

[209] Vetoshko PM Gusev NA Chepurnova DA Samoilova E V Zvezdin AK Korotaeva AA Rat magnetocardiography using a flux-gate sensor based on iron garnet films. Biomed Eng. (2016) 50:237. 10.1007/s10527-016-9628-9

[210] Sutter JU Lewis O Robinson C McMahon A Boyce R Bragg R Recording the heart beat of cattle using a gradiometer system of optically pumped magnetometers. Comput Electron Agric. (2020) 177(June):105651 (1–8). 10.1016/j.compag.2020.105651

[211] Park J-W Leithäuser B Hill P Jung F. Resting magnetocardiography predicts 3-year mortality in patients presenting with acute chest pain without ST segment elevation. Ann Noninvasive Electrocardiol. (2008) 13(2):171–9. 10.1111/j.1542-474X.2008.00217.x 18426443 PMC6932191

[212] Park J-W Hill PM Chung N Hugenholtz PG Jung F. Magnetocardiography predicts coronary artery disease in patients with acute chest pain. Ann Noninvasive Electrocardiol. (2005) 10(3):312–23. 10.1111/j.1542-474X.2005.00634.x 16029382 PMC6932599

[213] Hailer B Chaikovsky I Auth-Eisernitz S Schäfer H Steinberg F Grönemeyer DHW. Magnetocardiography in coronary artery disease with a new system in an unshielded setting. Clin Cardiol. (2003) 26(10):465–71. 10.1002/clc.4960261007 14579917 PMC6654687

[214] Van Leeuwen P Hailer B Lange S Gronemeyer D. Spatial distribution of repolarization times in patients with coronary artery disease. Pacing Clin Electrophysiol. (2003) 26(8):1706–14. 10.1046/j.1460-9592.2003.t01-1-00256.x 12877704

[215] Hailer B Chaikovsky I Auth-Eisernitz S Schäfer H Van Leeuwen P. The value of magnetocardiography in patients with and without relevant stenoses of the coronary arteries using an unshielded system. Pacing Clin Electrophysiol. (2005) 28(1):8–16. 10.1111/j.1540-8159.2005.09318.x 15660796

[216] Hailer B Van Leeuwen P Chaikovsky I Auth-Eisernitz S Schafer H Gronemeyer D. The value of magnetocardiography in the course of coronary intervention. Ann Noninvasive Electrocardiol. (2005) 10(2):188–96. 10.1111/j.1542-474X.2005.05625.x 15842431 PMC6932285

[217] Brazdeikis A Taylor AA Mahmarian JJ Xue Y CC. Comparison of magnetocardiograms acquired in unshielded clinical environment at rest, during and after exercise and in conjunction with myocardial perfusion imaging. In: Nowak H Haueisen J Gießler F Huonker R, editors. Biomag 2002, 13th international conference on biomagnetism. Berlin: VDE Verlag GMBH. (2002). p. 530–2.

[218] Sosnytskyy V Chaikovsky I Stadnyuk L Miasnykov G Kazmirchyk A Sosnytska T Magnetocardiography capabilities in myocardium injuries diagnosis. World J Cardiovasc Dis. (2013) 03(05):380–8. 10.4236/wjcd.2013.35059

[219] Sosnytska TV. Clinical application of magnetic mapping. Likars’ka Sprav. (2011) (1–2):29–47. Available at: http://www.ncbi.nlm.nih.gov/pubmed/21954633 (Accessed February 20, 2017). 21954633

[220] Brala D Thevathasan T Grahl S Barrow S Violano M Bergs H Application of magnetocardiography to screen for inflammatory cardiomyopathy and monitor treatment response. J Am Heart Assoc. (2023) 12(4):e027619. 10.1161/JAHA.122.027619 36744683 PMC10111485

[221] Kawakami S Takaki H Hashimoto S Kimura Y Nakashima T Aiba T Utility of high-resolution magnetocardiography to predict later cardiac events in nonischemic cardiomyopathy patients with normal QRS duration. Circ J. (2017) 81(1):44–51. 10.1253/circj.CJ-16-0683 27853097

[222] Brazdeikis A Chu CW Cherukuri P Litovsky S Naghavi M. Changes in magnetocardiogram patterns of infarcted-reperfused myocardium after injection of superparamagnetic contrast media. Neurol Clin Neurophysiol. (2004) 2004:16. Available at: http://www.ncbi.nlm.nih.gov/pubmed/16012681 (Accessed February 20, 2017). 16012681

[223] Padhye NS Brazdeikis A Verklan MT. Monitoring fetal development with magnetocardiography. Conf Proc IEEE Eng Med Biol Soc. (2004) 2004:3609–10. 10.1109/IEMBS.2004.1404014 17271072

[224] Verklan MT Padhye NS Brazdeikis A. Analysis of fetal heart rate variability obtained by magnetocardiography. J Perinat Neonatal Nurs. (2006) 20(4):343–8. 10.1097/00005237-200610000-00014 17310675

[225] Brisinda D Bottelli G Napolitano C Priori SG Fenici R. Magnetocardiographic findings and follow-up in an asymptomatic Brugada patient. Effects of flecainide and of exercise tests. Int Congr Ser. (2007) 1300:459–62. 10.1016/j.ics.2006.12.079

[226] Brisinda D Venuti A Sorbo AR Fenici R. Magnetocardiographic demonstration of complex ventricular preexcitation resulting in ablation failure. Int J Cardiol. (2013) 168(5):5046–8. 10.1016/j.ijcard.2013.07.215 23932861

[227] Hart G. Biomagnetometry: imaging the heart's magnetic field. Br Heart J. (1991) 65(2):61–2. 10.1136/hrt.65.2.61 1867947 PMC1024491

[228] Kim D Ahn H. Current status and future of cardiac mapping in atrial fibrillation. In: Choi JI, editor. Atrial fibrillation—basic research and clinical applications. Seul: InTech (2012). p. 93–124. 10.5772/25905

[229] Mäntynen V Vitikainen AM Koskinen R Mäkijärvi M Toivonen L Montonen J. Magnetocardiography is sensitive to differences in inter-atrial conduction in patients with paroxysmal lone atrial fibrillation. Int Congr Ser. (2007) 1300:508–11. 10.1016/j.ics.2006.12.081

[230] Sato Y Yoshida K Ogata K Inaba T Tada H Sekiguchi Y An increase in right atrial magnetic strength is a novel predictor of recurrence of atrial fibrillation after radiofrequency catheter ablation. Circ J. (2012) 76(7):1601–8. 10.1253/circj.CJ-11-1419 22473455

[231] Tsukada K Miyashita T Kandori A Mitsui T Terada Y Sato M An iso-integral mapping technique using magnetocardiogram, and its possible use for diagnosis of ischemic heart disease. Int J Card Imaging. (2000) 16(1):55–66. 10.1023/A:1006376326755 10832626

[232] Yoshida K Ogata K Inaba T Nakazawa Y Ito Y Yamaguchi I Ability of magnetocardiography to detect regional dominant frequencies of atrial fibrillation. J Arrhythmia. (2015) 31(6):345–51. 10.1016/j.joa.2015.05.003 PMC4672036 26702313

[233] Yoshida N Inden Y Uchikawa T Kamiya H Kitamura K Shimano M Novel transitional zone index allows more accurate differentiation between idiopathic right ventricular outflow tract and aortic sinus cusp ventricular arrhythmias. Hear Rhythm. (2011) 8(3):349–56. 10.1016/j.hrthm.2010.11.023 21078412

[234] Lehto M Jurkko R Parikka H Mäntynen V Väänänen H Montonen J Reversal of atrial remodeling after cardioversion of persistent atrial fibrillation measured with magnetocardiography. Pacing Clin Electrophysiol. (2009) 32(2):217–23. 10.1111/j.1540-8159.2008.02205.x 19170911

[235] Inaba T Nakazawa Y Yoshida K Kato Y Hattori A Kimura T Routine clinical heart examinations using SQUID magnetocardiography at University of Tsukuba Hospital. Supercond Sci Technol. (2017) 30(11):114003. 10.1088/1361-6668/aa8c26

[236] Jurkko R Mäntynen V Lehto M Tapanainen JM Montonen J Parikka H Interatrial conduction in patients with paroxysmal atrial fibrillation and in healthy subjects. Int J Cardiol. (2010) 145(3):455–60. 10.1016/j.ijcard.2009.05.064 19545922

[237] Taccardi B. Distribution of heart potentials on dog's thoracic surface. Circ Res. (1962) 11:862–9. 10.1161/01.RES.11.5.862 13980114

[238] Taccardi B. Distribution of heart potentials on the thoracic surface of normal human subjects. Circ Res. (1963) 12:341–52. 10.1161/01.RES.12.4.341 13980115

[239] Ramanathan C Ghanem RN Jia P Ryu K Rudy Y. Noninvasive electrocardiographic imaging for cardiac electrophysiology and arrhythmia. Nat Med. (2004) 10(4):422–8. 10.1038/nm1011 15034569 PMC1950745

[240] Berger T Fischer G Pfeifer B Modre R Hanser F Trieb T Single-beat noninvasive imaging of cardiac electrophysiology of ventricular pre-excitation. J Am Coll Cardiol. (2006) 48(10):2045–52. 10.1016/j.jacc.2006.08.019 17112994

[241] Revishvili AS Wissner E Lebedev DS Lemes C Deiss S Metzner A Validation of the mapping accuracy of a novel non-invasive epicardial and endocardial electrophysiology system. Europace. (2015) 17(8):1282–8. 10.1093/europace/euu339 25643987 PMC4535554

[242] Shah AJ Hocini M Pascale P Roten L Komatsu Y Daly M Body surface electrocardiographic mapping for non-invasive identification of arrhythmic sources. Arrhythmia Electrophysiol Rev. (2013) 2(1):16. 10.15420/aer.2013.2.1.16 PMC4711575 26835035

[243] Shah A Hocini M Haissaguerre M Jaïs P. Non-invasive mapping of cardiac arrhythmias. Curr Cardiol Rep. (2015) 17(8):60. 10.1007/s11886-015-0616-6 26072438

[244] Haïssaguerre M Hocini M Cheniti G Duchateau J Sacher F Puyo S Localized structural alterations underlying a subset of unexplained sudden cardiac death. Circ Arrhythmia Electrophysiol. (2018) 11(7):1–12. 10.1161/CIRCEP.117.006120 PMC7661047 30002064

[245] Her AY Shin ES Zhou Q Wierzbinski J Vidal-Lopez S Saleh A Magnetocardiography detects left atrial dysfunction in paroxysmal atrial fibrillation. Clin Hemorheol Microcirc. (2019) 72(4):353–63. 10.3233/CH-180528 30958336

[246] Aita S Ogata K Yoshida K Inaba T Kosuge H Machino T Noninvasive mapping of premature ventricular contractions by merging magnetocardiography and computed tomography. JACC Clin Electrophysiol. (2019) 5(10):1144–57. 10.1016/j.jacep.2019.06.010 31648739

[247] Lombardi G Sorbo AR Guida G La Brocca L Fenici R Brisinda D. Magnetocardiographic classification and non-invasive electro-anatomical imaging of outflow tract ventricular arrhythmias in recreational sport activity practitioners. J Electrocardiol. (2018) 51(3):433–9. 10.1016/j.jelectrocard.2018.02.004 29486898

[248] Guida G Sorbo AR Fenici R Brisinda D. Predictive value of unshielded magnetocardiographic mapping to differentiate atrial fibrillation patients from healthy subjects. Ann Noninvasive electrocardiol. (2018) 23(6):e12569. 10.1111/anec.12569 29947446 PMC6931487

[249] Ito Y Shiga K Yoshida K Ogata K Kandori A Inaba T Development of a magnetocardiography-based algorithm for discrimination between ventricular arrhythmias originating from the right ventricular outflow tract and those originating from the aortic sinus cusp: a pilot study. Hear Rhythm. (2014) 11(9):1605–12. 10.1016/j.hrthm.2014.05.032 24887136

[250] Lau S Petković B Haueisen J. Optimal magnetic sensor vests for cardiac source imaging. Sensors. (2016) 16(6):1–17. 10.3390/s16060754 PMC4934180 27231910

[251] Lombardi G Sorbo AR Fenici R Brisinda D. Phantom assessment of unshielded magnetocardiography repeatability, precision and accuracy in electric sources localization. Ann Hear. (2017) 1(2):35–40. 10.36959/652/388

[252] Khan MA Sun J Li B Przybysz A Kosel J. Magnetic sensors—a review and recent technologies. Eng Res Express. (2021) 3(2):22005. 10.1088/2631-8695/ac0838

[253] Murzin D. Ultrasensitive magnetic field sensors for biomedical applications. Sensors. (2020) 20:1569. 10.3390/s20061569 32168981 PMC7146409

[254] Wu YW Lin LC Tseng WK Bin LY Kao HL Lin MS QTc heterogeneity in rest magnetocardiography is sensitive to detect coronary artery disease: in comparison with stress myocardial perfusion imaging. Acta Cardiol Sin. (2014) 30(5):445–54. 10.1371/journal.pone.0133192 27122818 PMC4834957

[255] Shin E-S Chung J-H Park SG Saleh A Lam Y-Y Bhak J Comparison of exercise electrocardiography and magnetocardiography for detection of coronary artery disease using ST-segment fluctuation score. Clin Hemorheol Microcirc. (2019) 73(2):283–91. 10.3233/CH-180485 30775972

[256] Chaikovsky I Primin M Nedayvoda I Verba A Mjasnikov G Kazmirchyk A Magnetocardiographic polar map image reveal regional wall motion abnormalities: comparison study with stress-echocardiography. J Am Coll Cardiol. (2017) 70(16):C88. 10.1016/j.jacc.2017.07.309

[257] Desai L Wakai R Tsao S Strasburger J Gotteiner N Patel A. Fetal diagnosis of KCNQ1-variant long QT syndrome using fetal echocardiography and magnetocardiography. Pacing Clin Electrophysiol. (2020) 43(4):430–3. 10.1111/pace.13900 32168391 PMC7166171

[258] Gapelyuk A Schirdewan A Fischer R Wessel N. Cardiac magnetic field mapping quantified by Kullback–Leibler entropy detects patients with coronary artery disease. Physiol Meas. (2010) 31(10):1345–54. 10.1088/0967-3334/31/10/004 20720289

[259] Huang X Hua N Tang F Zhang S. Effectiveness of magnetocardiography to identify patients in need of coronary artery revascularization: a cross-sectional study. Cardiovasc Diagn Ther. (2020) 10(4):831–40. 10.21037/cdt-20-121 32968638 PMC7487377

[260] Mooney JW Ghasemi-Roudsari S Banham ER Symonds C Pawlowski N Varcoe BTH. A portable diagnostic device for cardiac magnetic field mapping. Biomed Phys Eng Express. (2017) 3(1):015008. 10.1088/2057-1976/3/1/015008

[261] Fenici R Brisinda D. Is there any place for magnetocardiographic imaging in the era of robotic ablation of cardiac arrhythmias? Funct Imaging Model Hear. (2007) 4466;230–9. 10.1007/978-3-540-72907-5_24

[262] Ghasemi-Roudsari S Al-Shimary A Varcoe B Byrom R Kearney L Kearney M. A portable prototype magnetometer to differentiate ischemic and non-ischemic heart disease in patients with chest pain. PLoS One. (2018) 13(1):1–10. 10.1371/journal.pone.0191241 PMC5774725 29351337

[263] Goodacre S Walters SJ Qayyum H Coffey F Carlton E Coats T Diagnostic accuracy of the magnetocardiograph for patients with suspected acute coronary syndrome. Emerg Med J. (2021) 38(1):47–52. 10.1136/emermed-2020-210396 33051274

[264] Beadle R McDonnell D Ghasemi-Roudsari S Unitt L Parker SJ Varcoe BTH. Assessing heart disease using a novel magnetocardiography device. Biomed Phys Eng Express. (2021) 7(2):25018. 10.1088/2057-1976/abe5c5 33578399

[265] Lachlan T He H Sharma K Khan J Rajappan K Morley-Davies A MAGNETO cardiography parameters to predict future sudden cardiac death (MAGNETO-SCD) or ventricular events from implantable cardioverter defibrillators: study protocol, design and rationale. BMJ Open. (2020) 10(10):1–7. 10.1136/bmjopen-2020-038804 PMC7552867 33040013

[266] Lachlan T He H Miller A Chandan N Siddiqui S Beadle R Feasibility of novel unshielded portable magnetocardiography: insights from the prospective multicenter MAGNETO-SCD trial. Heart Rhythm. (2023) 20:475–7. 10.1016/j.hrthm.2022.12.020 36549632

[267] Zhu K Shah AM Berkow J Kiourti A. Miniature coil array for passive magnetocardiography in non-shielded environments. IEEE J Electromagn RF Microwaves Med Biol. (2021) 5(2):124–31. 10.1109/JERM.2020.3019891

[268] Wang Z Zhu K Kaur A Recker R Yang J Kiourti A. Quantifying cognitive workload using a non-contact magnetocardiography (MCG) wearable sensor. Sensors. (2022) 22(23):1–12. 10.1109/JSEN.2022.3222553 PMC9735863 36501816

[269] Lin GG Scott JG. A compact, high performance atomic magnetometer for biomedical applications. Phys Med Biol. (2013) 100(2):130–4. 10.1088/0031-9155/58/22/8153 PMC3971838 24200837

[270] Tierney TM Holmes N Mellor S López JD Roberts G Hill RM Optically pumped magnetometers: from quantum origins to multi-channel magnetoencephalography. Neuroimage. (2019) 199(December 2018):598–608. 10.1016/j.neuroimage.2019.05.063 31141737 PMC6988110

[271] Kominis IK Kornack TW Allred JC Romalis MV. A subfemtotesla multichannel atomic magnetometer. Nature. (2003) 422(6932):596–9. 10.1038/nature01484 12686995

[272] Weis A Wynands R Fenici R Bison G. Dynamical MCG mapping with an atomic vapor magnetometer. Neurol Clin Neurophysiol. (2004) 2004:38. 16012670

[273] Strand S Lutter W Strasburger JF Shah V Baffa O Wakai RT. Low-cost fetal magnetocardiography: a comparison of superconducting quantum interference device and optically pumped magnetometers. J Am Heart Assoc. (2019) 8(16):1–10. 10.1161/JAHA.119.013436 PMC6759914 31394997

[274] Strand S Strasburger JF Cuneo BF Wakai RT. Complex and novel arrhythmias precede stillbirth in fetuses with de novo long QT syndrome. Circ Arrhythm Electrophysiol. (2020) 13(5):427–34. 10.1161/CIRCEP.119.008082 PMC7241276 32421437

[275] Fenici R Bison G Wynands R. Comparison of magnetocardiographic mapping with SQUID-based and laser-pumped magnetometers in normal subjects. Biomed Tech Band 48. (2004) 48(Ergänzungsband 2):192–4.

[276] Pena ME Pearson CL Goulet MP Kazan VM DeRita AL Szpunar SM A 90-second magnetocardiogram using a novel analysis system to assess for coronary artery stenosis in emergency department observation unit chest pain patients. IJC Hear Vasc. (2020) 26(100466):1–7. 10.1016/j.ijcha.2019.100466 PMC6956743 31956695

[277] Yang Y Xu M Liang A Yin Y Ma X Gao Y A new wearable multichannel magnetocardiogram system with a SERF atomic magnetometer array. Sci Rep. (2021) 11(1):1–12. 10.1038/s41598-020-79139-8 33692397 PMC7970947

[278] Fenici R Mashkar R Brisinda D. Performance of miniature scalar atomic magnetometers for magnetocardiography in an unshielded hospital laboratory for clinical electrophysiology. Eur Heart J. (2020) 41(Suppl_2):386. 10.1093/ehjci/ehaa946.0386

[279] Zhang R Mhaskar R Smith K Prouty M. Portable intrinsic gradiometer for ultra-sensitive detection of magnetic gradient in unshielded environment. Appl Phys Lett. (2020) 116(14):143501 10.1063/5.0004746

[280] Limes ME Foley EL Kornack TW Caliga S McBride S Braun A Portable magnetometry for detection of biomagnetism in ambient environments. Phys Rev Appl. (2020) 14(1):011002-1–6. 10.1103/PhysRevApplied.14.011002

[281] Zhang R Xiao W Ding Y Feng Y Peng X Shen L Recording brain activities in unshielded Earth's field with optically pumped atomic magnetometers. Sci Adv. (2020) 6(24):1–9. 10.1126/sciadv.aba8792 PMC7292643 32582858

[282] Perry AR Bulatowicz MD Larsen M Walker TG Wyllie R. All-optical intrinsic atomic gradiometer with sub-20 fT/cm/√Hz sensitivity in a 22 µT earth-scale magnetic field. Opt Express. (2020) 28(24):36696. 10.1364/OE.408486 33379758

[283] Clancy RJ Gerginov V Alem O Becker S Knappe S. A study of scalar optically-pumped magnetometers for use in magnetoencephalography without shielding. Phys Med Biol. (2021) 66(17):175030. 10.1088/1361-6560/ac18fb PMC9273178 34325403

[284] Petrenko M Vershovskii A. Towards a practical implementation of a single-beam all-optical non-zero-field magnetic sensor for magnetoencephalographic complexes. Sensors. (2022) 22(9862):1–12. 10.3390/s22249862 PMC9784752 36560230

[285] Xiao W Sun C Shen L Feng Y Liu M Wu Y A movable unshielded magnetocardiography system. Sci Adv. (2023) 9(13):eadg1746. 10.1126/sciadv.adg1746 36989361 PMC10058232

[286] Kim YJ Savukov I Newman S. Magnetocardiography with a 16-channel fiber-coupled single-cell Rb optically pumped magnetometer. Appl Phys Lett. (2019) 114(14):143702 (1–4). 10.1063/1.5094339

[287] Sheng D Perry AR Krzyzewski SP Geller S Kitching J Knappe S. A microfabricated optically-pumped magnetic gradiometer. Appl Phys Lett. (2017) 110(3):031106 (1–4). 10.1063/1.4974349 28179732 PMC5250637

[288] Sulai IA Deland ZJ Bulatowicz MD Wahl CP Wakai RT Walker TG. Characterizing atomic magnetic gradiometers for fetal magnetocardiography. Rev Sci Instrum. (2019) 90(8):085003-1. 10.1063/1.5091007 31472627 PMC6690843

[289] Dale MW Morley GW. Medical applications of diamond magnetometry: commercial viability. Appl Phys. (2017):1–10. 10.48550/arXiv.1705.01994

[290] Petrini G Moreva E Bernardi E Traina P Tomagra G Carabelli V Is a quantum biosensing revolution approaching? Perspectives in NV-assisted current and thermal biosensing in living cells. Adv Quantum Technol. (2020) 3(12):1–20. 10.1002/qute.202000066

[291] Chatzidrosos G Wickenbrock A Bougas L Leefer N Wu T Jensen K Miniature cavity-enhanced diamond magnetometer. Phys Rev Appl. (2017) 8(4):6–10. 10.1103/PhysRevApplied.8.044019

[292] Jensen K Kehayias P Budker D. Magnetometry with nitrogen-vacancy centers in diamond. Smart Sensors Meas Instrum. (2017) 19(September):553–76. 10.1007/978-3-319-34070-8_18

[293] Graham SM Rahman ATMA Munn L Patel RL Newman AJ Stephen CJ Fiber-coupled diamond magnetometry with an unshielded 15 pT/√Hz sensitivity. Phys Rev Applied. (2022) (1):1–20. 10.48550/arXiv.2211.09170

[294] Barry JF Turner MJ Schloss JM Glenn DR Song Y Lukin MD Optical magnetic detection of single-neuron action potentials using quantum defects in diamond. Proc Natl Acad Sci U S A. (2016) (18):201601513. 10.1073/pnas.1601513113 PMC5150388 27911765

[295] Zhang C Zhang J Widmann M Benke M Kübler M Dasari D Optimizing NV magnetometry for magnetoneurography and magnetomyography applications. Front Neurosci. (2023) 16:1–16. 10.3389/fnins.2022.1034391 PMC9885266 36726853

[296] Gusev NA Vetoshko PM Kuzmichev AN Chepurnova DA Samoilova E V Zvezdin AK Ultra-sensitive vector magnetometer for magnetocardiographic mapping. Biomed Eng. (2017) 51(3):157–61. 10.1007/s10527-017-9705-8

[297] Ma J Yang Z Uchiyama T. Development of high resolution programmable oversampling MI sensor system with 32-bit ADC for multi-channel bio-magnetic measurements. AIP Adv. (2019) 9(12):125243 (1–6). 10.1063/1.5129842

[298] Shirai Y Hirao K Shibuya T Okawa S Hasegawa Y Adachi Y Magnetocardiography using a magnetoresistive sensor array. Int Heart J. (2019) 60(1):50–4. 10.1536/ihj.18-002 30464123

[299] Kurashima K Kataoka M Nakano T Fujiwara K Kato S Nakamura T Development of magnetocardiograph without magnetically shielded room using high-detectivity TMR sensors. Sensors. (2023) 23(2):646 (1–18). 10.3390/s23020646 36679442 PMC9866167

[300] Adachi Y Oyama D Terazono Y Hayashi T Shibuya T Kawabata S. Calibration of room temperature magnetic sensor array for biomagnetic measurement. IEEE Trans Magn. (2019) 55(7):1–6. 10.1109/TMAG.2019.2895355

[301] Shen HM Hu L Fu X. Integrated giant magnetoresistance technology for approachable weak biomagnetic signal detections. Sensors. (2018) 18(1):1–20. 10.3390/s18010148 PMC5795475 29316670

[302] Wu K Tonini D Liang S Saha R Chugh VK Wang J-P. Giant magnetoresistance biosensors in biomedical applications. ACS Appl Mater Interfaces. (2022) 14(8):9945–69. 10.1021/acsami.1c20141 35167743 PMC9055838

[303] Zhang J Liu J Zhang Q Filippov DA Li K Wu J High-resolution magnetic sensors in ferrite/piezoelectric heterostructure with giant magnetodielectric effect at zero bias field. Rev Sci Instrum. (2021) 92(4):1ENG. 10.1063/5.0035059 34243376

[304] Thompson MD Ben Shalom M Geim AK Matthews AJ White J Melhem Z Graphene-based tunable SQUIDs. Appl Phys Lett. (2017) 110(16):162602 (1–5). 10.1063/1.4981904

[305] Li T Gallop JC Hao L Romans EJ. Scalable, tunable Josephson junctions and DC SQUIDs based on CVD graphene. IEEE Trans Appl Supercond. (2019) 29(5):1–4. 10.1109/TASC.2019.2897999

[306] Indolese DI Karnatak P Kononov A Delagrange R Haller R Wang L Compact SQUID realized in a double-layer graphene heterostructure. Nano Lett. (2020) 20(10):7129–35. 10.1021/acs.nanolett.0c02412 32872789

[307] Kim K Lee YH Kwon H Kim JM Kim IS Park YK. Averaging algorithm based on data statistics in magnetocardiography. Neurol Clin Neurophysiol. (2004) 2004:42. Available at: http://www.ncbi.nlm.nih.gov/pubmed/16015714 (Accessed February 20, 2017). 16015714

[308] Dang-Ting L Ye T Yu-Feng R Hong-Wei Y Li-Hua Z Qian-Sheng Y A novel filter scheme of data processing for SQUID-based magnetocardiogram. Chinese Phys Lett. (2008) 25(7):2714. 10.1088/0256-307X/25/7/105

[309] Comani S Mantini D Alleva G Di Luzio S Romani GL. Optimal filter design for shielded and unshielded ambient noise reduction in fetal magnetocardiography. Phys Med Biol. (2005) 50(23):5509–21. 10.1088/0031-9155/50/23/006 16306648

[310] Mensah-brown NA Lutter WJ Comani S Strasburger JF Wakai RT. Independent component analysis of normal and abnormal rhythm in twin pregnancies. Physiol Meas. (2011) 32(1):51–64. 10.1088/0967-3334/32/1/004 21098910 PMC3003612

[311] Tiporlini V Alameh K. Optical magnetometer employing adaptive noise cancellation for unshielded magnetocardiography. Univers J Biomed Eng. (2013) 1(1):16–21. 10.13189/ujbe.2013.010104

[312] Bakharev AA. High balance gradiometer. Vol. 1. United States Patent Office. US 2003/0141868A1(2003). p. 1–7.

[313] Mohsen A Al-Mahdawi M Fouda MM Oogane M Ando Y Fadlullah ZM. AI aided noise processing of spintronic based IoT sensor for magnetocardiography application. In: *ICC 2020–2020 IEEE International Conference on Communications (ICC)*. Ithaca, New York: (2020). p. 1–6.

[314] Parimita SP Sengottuvel S Patel R Mani A Gireesan K. A feasibility study to measure magnetocardiography (MCG) in unshielded environment using first order gradiometer. Biomed Signal Process Control. (2020) 55:101664. 10.1016/j.bspc.2019.101664

[315] Mariyappa N Sengottuvel S Parasakthi C Gireesan K Janawadkar MP Radhakrishnan TS Baseline drift removal and denoising of MCG data using EEMD: role of noise amplitude and the thresholding effect. Med Eng Phys. (2014) 36(10):1266–76. 10.1016/j.medengphy.2014.06.023 25074650

[316] Liao Y He C Guo Q. Denoising of magnetocardiography based on improved variational mode decomposition and interval thresholding method. Symmetry. (2018) 10(7):269 (1–14). 10.3390/sym10070269

[317] Pyragius T Jensen K. A high performance active noise control system for magnetic fields. Rev Sci Instrum. (2021) 92(12):1–8. 10.1063/5.0062650 34972433

[318] Takiya T Uchiyama T. Development of active shielding-type MI gradiometer and application for magnetocardiography. IEEE Trans Magn. (2017) 53(11):1–4. 10.1109/TMAG.2017.2726111

[319] Petrenko M Vershovskii A. Towards a practical implementation of a single-beam all-optical non-zero-field magnetic sensor for magnetoencephalographic complexes. Sensors. (2022) 22(24):9862 (1–12). 10.3390/s22249862 36560230 PMC9784752

[320] Wissner E Revishvili A Metzner A Tsyganov A Kalinin V Lemes C Noninvasive epicardial and endocardial mapping of premature ventricular contractions. Europace. (2017) 19(5):843–9. 10.1093/europace/euw103 27207812 PMC5437699

[321] Tsyganov A Wissner E Metzner A Mironovich S Chaykovskaya M Kalinin V Mapping of ventricular arrhythmias using a novel noninvasive epicardial and endocardial electrophysiology system. J Electrocardiol. (2018) 51(1):92–8. 10.1016/j.jelectrocard.2017.07.018 28912073

[322] Bear LR Bouhamama O Cluitmans M Duchateau J Walton RD Abell E Advantages and pitfalls of noninvasive electrocardiographic imaging. J Electrocardiol. (2019) 57:S15–20. 10.1016/j.jelectrocard.2019.08.007 31477238

[323] Duchateau J Sacher F Pambrun T Derval N Chamorro-Servent J Denis A Performance and limitations of noninvasive cardiac activation mapping. Hear Rhythm. (2019) 16(3):435–42. 10.1016/j.hrthm.2018.10.010 30385382

[324] Mäntynen V Konttila T Stenroos M. Investigations of sensitivity and resolution of ECG and MCG in a realistically shaped thorax model. Phys Med Biol. (2014) 59(23):7141–58. 10.1088/0031-9155/59/23/7141 25365547

[325] Scherlag BJ Yamanashi WS Hou Y Jacobson JI Jackman WM Lazzara R. Magnetism and cardiac arrhythmias. Cardiol Rev. (2004) 12(2):85–96. 10.1097/01.crd.0000094029.10223.2f 14766023

[326] Yu L Dyer JW Scherlag BJ Stavrakis S Sha Y Sheng X The use of low-level electromagnetic fields to suppress atrial fibrillation. Hear Rhythm. (2015) 12(4):809–17. 10.1016/j.hrthm.2014.12.022 25533588

[327] Wang S Zhou X Huang B Wang Z Zhou L Wang M Noninvasive low-frequency electromagnetic stimulation of the left stellate ganglion reduces myocardial infarction-induced ventricular arrhythmia. Sci Rep. (2016) 6(January):1–9. 10.1038/srep30783 27470078 PMC4965791

[328] Armour JA. Potential clinical relevance of the “little brain” on the mammalian heart. Exp Physiol. (2008) 93(2):165–76. 10.1113/expphysiol.2007.041178 17981929

[329] McCraty R. *Exploring the role of the heart in human performance*. Vol. 2. (2015). Available at: https://www.heartmath.org. (last accessed 02 July 2023)

[330] Pedersen M Abbott DF Jackson GD. Wearable OPM-MEG: a changing landscape for epilepsy. Epilepsia. (2022) 63(11):2745–53. 10.1111/epi.17368 35841260 PMC9805039

[331] Roth BJ. Biomagnetism: the first sixty years. Sensors. (2023) 23(9):4215. 10.3390/s23094215 37177427 PMC10181075

[332] Park J-W Leithäuser B Vrsansky M Jung F. Dobutamine stress magnetocardiography for the detection of significant coronary artery stenoses—a prospective study in comparison with simultaneous 12-lead electrocardiography. Clin Hemorheol Microcirc. (2008) 39(1–4):21–32. 10.3233/CH-2008-1064 18503107

[333] Park J-W Shin E-S Ann SH Gödde M Park LS-I Brachmann J Validation of magnetocardiography versus fractional flow reserve for detection of coronary artery disease. Clin Hemorheol Microcirc. (2015) 59(3):267–81. 10.3233/CH-141912 25480934

[334] Hänninen H Takala P Korhonen P Oikarinen L Mäkijärvi M Nenonen J Features of ST segment and T-wave in exercise-induced myocardial ischemia evaluated with multichannel magnetocardiography. Ann Med. (2002) 34(2):120–9. 10.1080/07853890252953518 12108575

[335] Fenici R Brisinda D. From 3D to 4D imaging: is that useful for interventional cardiac electrophysiology? Annu Int Conf IEEE Eng Med Biol Soc. (2007) 2007:5996–9. 10.1109/IEMBS.2007.4353714 18003380

[336] Mäkelä T Pham QC Clarysse P Nenonen J Lötjönen J Sipilä O A 3-D model-based registration approach for the PET, MR and MCG cardiac data fusion. Med Image Anal. (2003) 7(3):377–89. 10.1016/S1361-8415(03)00012-4 12946476

[337] Nademanee K Chung F-P Sacher F Nogami A Nakagawa H Jiang C Long-term outcomes of Brugada substrate ablation: a report from BRAVO (Brugada ablation of VF substrate ongoing multicenter registry). Circulation. (2023) 147:1568–78. 10.1161/CIRCULATIONAHA.122.063367 36960730

[338] Romani GL Williamson SJ. Fourth international workshop on biomagnetism. Nuovo Cim D. (1983) 2(2):121–2. 10.1007/BF02455916

[339] Burghoff M Nenonen J Trahms L Katila T. Conversion of magnetocardiographic recordings between two different multichannel SQUID devices. IEEE Trans Biomed Eng. (2000) 47(7):869–75. 10.1109/10.846680 10916257

